# Applications of Light-Based 3D Bioprinting and Photoactive Biomaterials for Tissue Engineering

**DOI:** 10.3390/ma16237461

**Published:** 2023-11-30

**Authors:** Xueqin Zhang, Xin Zhang, Ying Li, Yuxuan Zhang

**Affiliations:** 1 College of Chemistry and Materials Engineering, Beijing Technology and Business University, Beijing 100048, China; 2 FuYang Sineva Materials Technology Co., LTD., Beijing 100176, China

**Keywords:** light-based 3D bioprinting, photopolymerization, hydrogel, photoactive biomaterials, biocompatibility, tissue engineering

## Abstract

The emergence of additive manufacturing, commonly referred to as 3D printing, has led to a revolution in the field of biofabrication. Numerous types of 3D bioprinting, including extrusion bioprinting, inkjet bioprinting, and lithography-based bioprinting, have been developed and have played pivotal roles in driving a multitude of pioneering breakthroughs in the fields of tissue engineering and regenerative medicine. Among all the 3D bioprinting methods, light-based bioprinting utilizes light to crosslink or solidify photoreactive biomaterials, offering unprecedented spatiotemporal control over biomaterials and enabling the creation of 3D structures with extremely high resolution and precision. However, the lack of suitable photoactive biomaterials has hindered the application of light-based bioprinting in tissue engineering. The development of photoactive biomaterials has only recently been expanded. Therefore, this review summarizes the latest advancements in light-based 3D bioprinting technologies, including the development of light-based bioprinting techniques, photo-initiators (PIs), and photoactive biomaterials and their corresponding applications. Moreover, the challenges facing bioprinting are discussed, and future development directions are proposed.

## 1. Introduction

**Figure 1:**
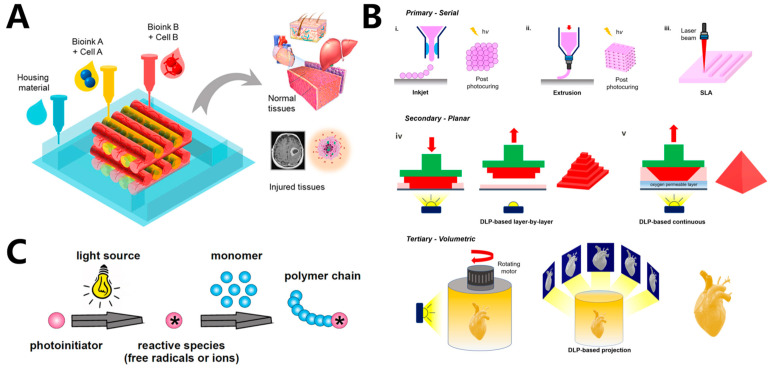
(**A**) 3D bioprinting structures for tissue engineering [12]. (**B**) Schematic illustration of light-based bioprinting technology, including inkjet bioprinting, extrusion bioprinting, and lithography-based bioprinting. i–iii: in primary configuration, structures are printed either dot by dot or line by line. iv–v: in secondary configuration, structures are printed layer by layer via DLP-based projection of patterns into a vat containing bio-ink. -In tertiary configuration, 3D structures are created volumetrically by projecting patterns into a rotating vat containing bio-inks [7]. (**C**) General mechanism of photopolymerization [35].

The incidence of vital human organ failure has significantly increased with the extended human lifespan. The emergence and development of tissue engineering provide a promising solution to these challenges and are considered to have provided an effective method for eventually achieving the regeneration of human tissues and organs in the future [1,2,3]. Hydrogels are ideal materials for tissue engineering, as they can be tailored to a variety of mechanical, chemical, and biological characteristics for cell adhesion, proliferation, and migration. The conventional tissue-engineering strategy entails seeding cells onto a porous hydrogel scaffold first. With subsequent in vivo culturing, these cells undergo proliferation and differentiation, ultimately leading to the construction of a biological substitute [4,5,6]. Dynamic reciprocity within a 3D microenvironment, which can simulate the extracellular matrix (ECM), is crucial for cell growth [7]. Thus, it is very important to control the biomaterials in 3D space precisely to fabricate scaffolds with adjustable mechanical, physical, and rheological characteristics that perfectly mimic ECM. Traditional techniques such as freeze-drying [8], electrospinning [9], and thermally induced phase separation [10] make fabricating hydrogel scaffolds with precisely controlled microstructures difficult. The advent of 3D bioprinting made it possible to construct complex organ and tissue-like structures accurately (Figure 1A) [11,12,13,14,15]. Using computer-aided design (CAD), 3D printing can build desired structures in a precise and reproducible way [16,17]. Three-dimensional bioprinting based on traditional 3D printing can integrate cells, biomaterials, and bioactive factors into user-set geometries, which makes it a powerful tool in the fabrication of complex biomimetic tissue [18,19], drug-testing models [20], disease models [21], surgical implants [22], and smart sensors [23].

Within decades, a variety of 3D bioprinting techniques have been developed, including extrusion bioprinting [24,25], inkjet bioprinting [26], stereolithography (SLA)-based bioprinting [27], digital-light-processing-based (DLP-based) bioprinting [28], and computed-axial-lithography-based (CAL-based) bioprinting [29]. Hydrogels employed in 3D printing are crosslinked using various strategies, including chemical and physical crosslinking, to achieve the required strength and stability for maintaining the fidelity and resolution of the printed structures [30]. Additionally, the crosslinking of hydrogels can provide adequate support for cell growth within the printed structures. Chemical crosslinking methods, such as azide–alkyne cycloaddition, hydrazide–aldehyde coupling, thiol-ene coupling, enzymatic crosslinking, and photocrosslinking, offer significant advantages in 3D bioprinting of tissues [30]. Covalent bonds formed through these methods tend to provide greater tunability and higher stability for the printed structures, making them ideal for creating bioprinted tissues. Physical crosslinking methods, including hydrogen bonds, hydrophobic interactions, and ionic interactions, are also employed for crosslinking bioprinted hydrogels [31]. However, hydrogels crosslinked through these non-covalent bonds tend to be less stable, which makes them unsuitable for long-term in vitro cultivation. Currently, many of the 3D bioprinting methods employ light to crosslink or solidify photoreactive bio-inks. Extrusion-based bioprinting can use light to crosslink bio-inks before, after, or during extrusion, while lithography bioprinting can use light to directly solidify bio-inks (Figure 1B). Using light in 3D bioprinting offers several advantages, including rapid reaction rates, minimal heat production, and spatiotemporal control of the reaction [32]. Light-based bioprinting can be realized through the photopolymerization of photosensitive materials. In contrast to conventional bio-inks, the bio-inks utilized in light-based 3D bioprinting need to be integrated with photoreactive moieties to enable fast and selective photopolymerization of the bio-inks (Figure 1C) [33]. Normally, UV light and visible light can both be used as light sources in photopolymerization. However, as UV light may induce genetic mutations and even lead to cell death, visible light sources are commonly employed in light-based bioprinting to ensure cell viability and avoid potential harm to cells [34]. Photo-initiators (PIs) are a key component of photosensitive bio-inks. When irradiated by light, PIs can be excited to generate active species and subsequently initiate the polymerization of biomaterials. Within the last two decades, PIs and biomaterials with excellent biocompatibility, bioactivity, and biodegradability, which are suitable to be used in light-based 3D bioprinting, have been developed [7]. Another advantage of light-based bioprinting is that photopolymerization reactions can occur within aqueous solutions under physiological conditions, which can significantly reduce the use of harsh and cytotoxic reagents. Thus, light-based bioprinting is exceptionally well-suited for applications involving cells.

Since light-based bioprinting has shown magnificent potential for the fabrication of complex human tissues and organs, this review summarizes the latest advancements in light-based 3D bioprinting technologies, including specific light-based bioprinting technologies, the development of PIs and biomaterials, and corresponding applications. Moreover, the challenges facing bioprinting were discussed, and future development directions were prospected.

## 2. Light-Based 3D Bioprinting Methods and Applications

Light-based bioprinting has advantages, such as high printing resolution, the capability to print various materials, a controllable microstructure, minimal damage to cells, strong controllability, and fast processing speed. Among various techniques, extrusion bioprinting, SLA, and DLP have reaped the greatest benefits from the practicality of photoactivated biomaterials so far. The ultimate objective of tissue engineering and regenerative medicine is to successfully construct human organs and tissues. However, the current state of technology falls short of fully achieving the regeneration of organ tissues. Light-based bioprinting uses light as a source of energy for manufacturing, which allows the precise manipulation of photocurable materials, growth factors, and cells, in terms of both space and time, to create intricate structures. Researchers are harnessing bioprinting techniques to emulate complex human tissues in controlled laboratory environments, thus offering promising potential for organ and tissue regeneration. Presently, there is a wealth of research focused on employing light-based 3D bioprinting for the regeneration of diverse tissues, including bone [36], skin [37], liver [38], heart [38,39], blood vessels [40], and so on. In this section, light-based bioprinting methods, including extrusion-based bioprinting, SLA, DLP, and computed axial lithography (CAL), will be introduced.

### 2.1. Light-Based Inkjet 3D Bioprinting

Inkjet bioprinting is derived from the commonly used 2D inkjet printing. Ink droplets are propelled out of a microscopic orifice via thermal or piezoelectric actuation and deposited drop by drop on the platform to fabricate 3D structures. The generated droplets are on the micrometer scale (10–50 μm in diameter) to ensure the printing resolution [41]. After being deposited on the printing platform, the droplet can be gelled simultaneously by physical and chemical (irradiation) processes, thus ensuring printing fidelity [42]. Inkjet bioprinting offers several advantages, including fast printing speed, high cell viability, and low cost. Additionally, the cell viability in the droplet remains high after printing. Furthermore, by incorporating multiple inkjet heads, the fabrication of 3D multimaterial or multicolor structures can be achieved, a feat that is quite challenging with SLA or DLP [43]. Therefore, inkjet printing has gained widespread attention in the field of tissue engineering [26,44,45]. Mugnaini et al. [46] synthesized photocrosslinkable methacrylic pullulan. Aqueous dispersions of methacrylated pullulan were used as the bio-ink. Inkjet printing demonstrated shorter printing times and higher flexibility in printable architectures. Nevertheless, inkjet printing also presents some limitations. Its printing process is often accompanied by the generation of satellite droplets, which negatively impacts the printing.

### 2.2. Light-Based Extrusion 3D Bioprinting

**Figure 2:**
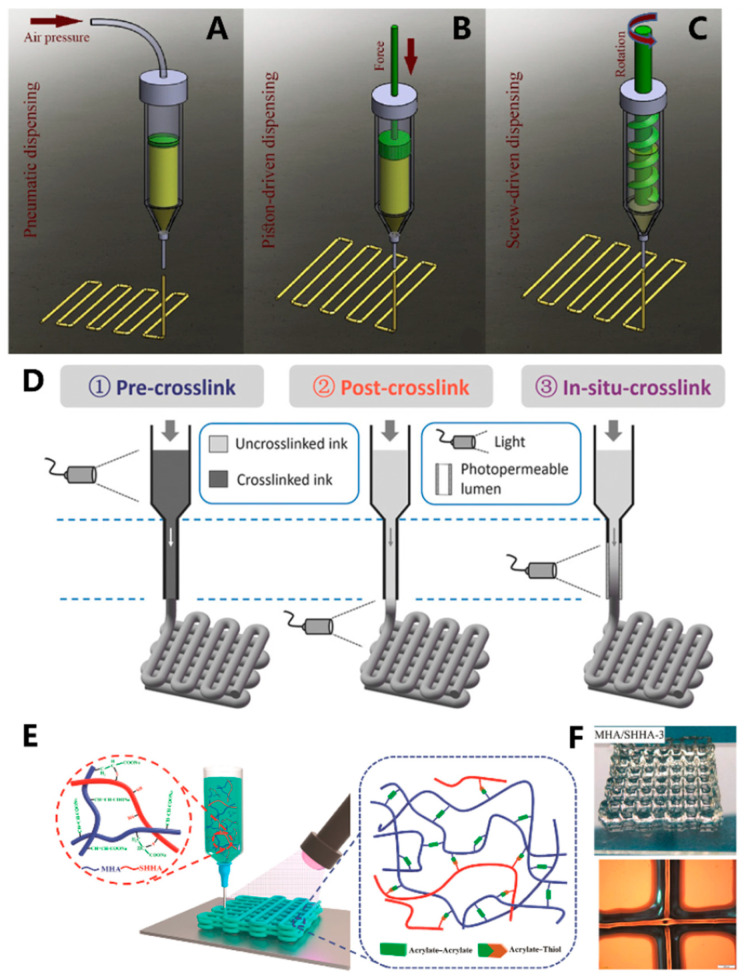
Schematic illustration of the three extrusion bioprinting methods: (**A**) pneumatic, (**B**) piston-driven, and (**C**) screw-driven dispensing method [49]; (**D**) schematic of three different crosslinking strategies for bioprinting HAMA inks, where crosslinking occurs before, after, or during extrusion [54]; (**E**) mechanism of the formation MHA/SHHA hydrogels [55]; (**F**) top: image of the printed MHA/SHHA hydrogel network, down: microscopy image of the printed MHA/SHHA hydrogel [55].

Due to its simplicity, versatility, reliable nature, and relatively cost-effectiveness, extrusion 3D printing stands as the most commonly used bioprinting technique for fabricating cell-laden hydrogel networks [32,47,48]. The printing process selectively deposits bio-inks, which are composed of cells, biomaterials, growth factors, and other components layer by layer on the printing platform. It can be categorized into pneumatic, piston-driven, and screw-driven dispensing (Figure 2A–C) [49]. Pneumatic dispensing uses air pressure to extrude bio-inks, while piston and screw-driven dispensings use vertical and rotational mechanical forces to extrude bio-inks, respectively. Bio-inks with viscosity ranging from 30 cP to 6 × 10^7^ cP are suitable for extrusion 3D bioprinting. As the extrusion process involves the extrusion of bio-inks from syringes with narrow nozzles or needles, bio-inks possessing shear-thinning characteristics are more favored for the extrusion process [50,51,52]. Nonetheless, extrusion-based printing does have its limitations. The printing resolution of extrusion-based printing is relatively low (>100 μm) when compared to other light-based 3D bioprinting [53]. Moreover, the shear forces produced during the extrusion process can result in reduced cell viability, which becomes particularly evident when higher-density cells are encapsulated.

Light-assisted extrusion bioprinting offers a way to address the shortcomings of conventional extrusion-based 3D printing. Irradiation can be applied during the printing process, after the completion of printing, or after the deposition of each extrusion layer (Figure 1B) [49]. The timing of light exposure depends primarily on the nature of the bio-ink and the stability of each layer. Ouyang et al. [54] utilized a photocurable HAMA (methacrylated hyaluronic acid, structure is shown in Figure S1)-based bio-ink containing mouse embryonic fibroblasts (NIH/3T3) as raw material for 3D bioprinting. They conducted pre-crosslinking (exposure before extrusion), post-crosslinking (exposure after extrusion), and in situ crosslinking (exposure during extrusion) strategies (Figure 2D). The results demonstrated that pre-crosslinked HAMA displayed reduced flowability through the printing nozzle due to prior crosslinking, leading to decreased cell viability (approximately 47%) caused by cell compression. Although post-extrusion light exposure improved cell viability, the low-viscosity HAMA bio-ink led to poor 3D structural formation. By replacing the printing nozzle with a transparent capillary and introducing light exposure during the extrusion process, the HAMA hydrogel can crosslink prior to deposition. The in situ crosslinking method effectively enhances the formability of the bio-ink, reduces the pressure exerted on cells during extrusion, and ultimately raises cell survival rates to above 95%. Wan et al. [55] fabricated a malleated sodium hyaluronate (MHA)/thiolated sodium hyaluronate (SHHA) hydrogel by simultaneous extrusion deposition and thiol-acrylate photopolymerization (Figure 2E). The obtained MHA/SHHA 3D structure showed good structural stability and high resolution (Figure 2F).

### 2.3. Suspension Bioprinting

**Figure 3:**
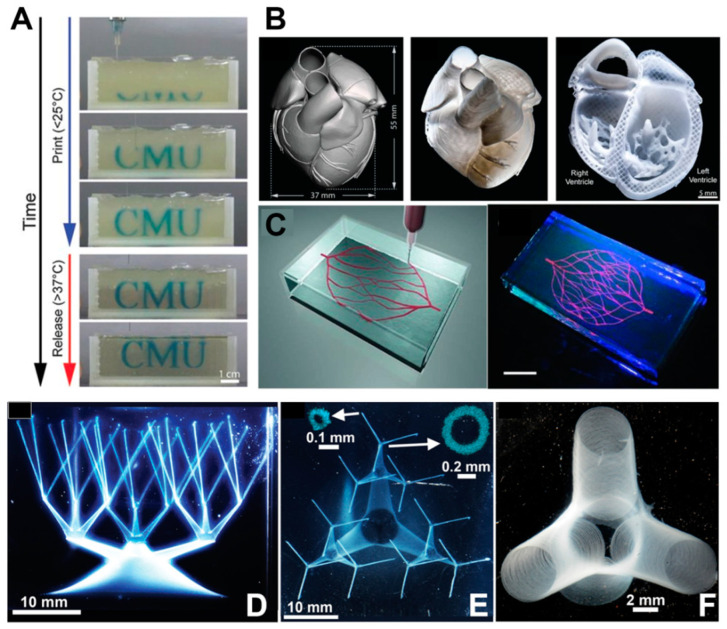
(**A**) Time-lapse sequence of 3D bioprinting of the letters “CMU” using FRESH v2.0 [55]; (**B**) Left: MRI-derived 3D human heart scaled to neonatal size. Middle: organ-scale FRESH 3D bioprinting of neonatal-scale human heart (middle image). Right: screw-driven dispensing method (right image) [55]; (**C**) Left: schematic illustration of the printing of a vascular structure using omnidirectional printing. Right: fluorescence photograph of the vascular structure printed using omnidirectional printing within a photo-polymerizable Pluronic F-127–diacrylate matrix, scale bar: 10 mm [57]. (**D**,**E**) Structures resembling hollow vessels featuring a wide range of sizes in both diameter and aspect ratio. Scale bar: 10 mm. Insets: Confocal cross-sections with a scale of 0.1 mm [56]. (**F**) An image depicting truncated vessels near a junction exhibited hollow tubes with slender walls, and the diameter of the vessel is about 100 μm, with a scale bar of 2 mm [56].

To address the issue of poor formability of bio-inks with low viscosity, Lee et al. developed the freeform reversible embedding of suspended hydrogels (FRESH) technology (Figure 3A) [55]. In this technique, bio-ink is extruded into a shear-thinning fluid bed, which can provide adequate support for shaping the bio-ink. The support material is solid at low shear stress and exhibits fluidity at high shear stress. Notably, at low levels of shear stress, the support material maintains its solidity, displaying high viscosity and resistance to deformation. However, as the shear stress increases, the support material becomes more fluid and exhibits reduced viscosity [56]. After the completion of printing, the support material is washed away. Complex structures such as heart valves and heart structures with high precision were fabricated using FRESH (Figure 3B). The printed ventricle exhibited synchronized contraction, and the wall thickness of the ventricle showed a 14% increase in thickness during contraction. Moreover, the heart structure was capable of electrical signal propagation. Wu et al. [57] employed omnidirectional freeform fabrication with sacrifice ink within a photopolymerizable Pluronic F-127–diacrylate matrix to fabricate 3D biomimetic microvascular networks arbitrary designs (Figure 3C). Bhattacharjee et al. [56] used a non-thixotropic granular Carbopol ETD 2020 polymer soft granular gel as the support medium. The medium exhibited local shear thinning and became fluidic near the extrusion nozzle without disturbing neighboring regions. When the nozzle moved away, the gel rapidly solidified. This allows for the fabrication of structures that were difficult to print before. Using this soft gel, the team was able to create intricate large 3D structures (including thin closed shells and hierarchically branched tubular networks) using a variety of materials such as silicones, hydrogels, colloids, and living cells (Figure 3D–F). These printed structures exhibited high fidelity and a high aspect ratio, demonstrating the potential of suspension bioprinting for tissue fabrication, particularly vascular structure fabrication.

### 2.4. Stereolithography, SLA

**Figure 4:**
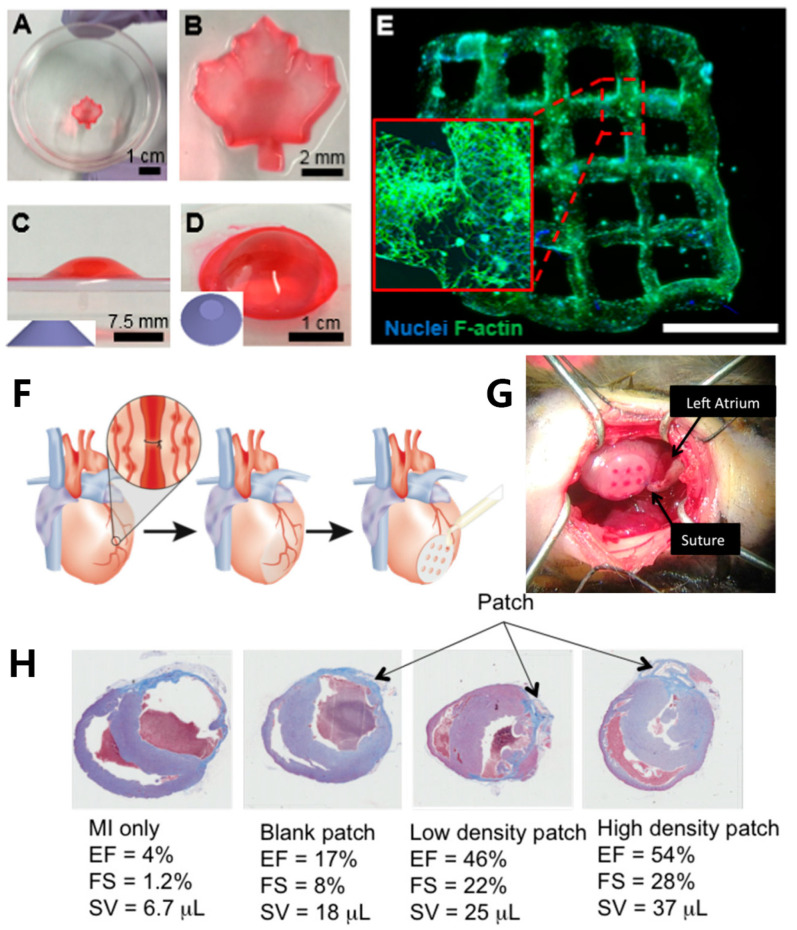
Maple-leaf pattern hydrogel networks fabricated by SLA: (**A**) top view of the hydrogel [59]; (**B**) magnified image of the printed hydrogel [59]; Images depicting the truncated cone structure from a lateral perspective (**C**) and an overhead view (**D**) [59]; (**E**) bioprinted NIH-3T3 cell-laden hydrogel sample cultured for 5 days, DAPI was used to stain nuclei (blue) and phalloidin 488 was used to stain F-actin (green), scale bar: 2 mm [59]; (**F**) schematic illustration of the in vivo murine myocardial infarction model and the placement of gel patch [61]; (**G**) image depicting the positioning of the patch onto the outer surface of the heart (epicardium) [61]; (**H**) histological pictures illustrating the state of each heart condition with different gel patches after 8 weeks [61].

SLA, one of the earliest commercialized 3D printing technologies, made its debut in bioprinting in 2004 when Boland’s team at Clemens University employed SLA to craft cell-encapsulated poly(ethylene glycol) diacrylate (PEGDA, Figure S3) porous tissue-engineering scaffolds [58]. This marked the inception of SLA’s application in the field of bioprinting. Compared with extrusion printing, where bio-ink within ladened cells is physically extruded on a printing platform, SLA uses a focused laser to selectively solidify the bio-ink layer by layer. As a result, SLA offers advantages, including high spatial resolution (20~50 μm), multiscalability, rapid printing speed of complex structures (lattice and patterned structures), and higher cell viability. Presently, SLA has garnered widespread attention for the fabrication of tissue-engineering scaffolds. Wang et al. [59] utilized an SLA printer equipped with a 500~600 nm laser bioprinted fibroblast-laden GelMA (methacrylated gelatin, Figure S4) hydrogels featuring complex structures (Figure 4A–D). Eosin Y was employed as the PI. The NIH-3T3 cell encapsulated within the hydrogel demonstrated robust viability and proliferated well to form 3D intercellular networks (Figure 4E). The results indicated that the Eosin Y/GelMA system was suitable for long-duration bioprinting and tissue regeneration. Lam et al. [60] reported a swine-derived chondrocyte-laden photopolymerized HAMA hydrogel network, which was bioprinted by SLA. After culturing for 14 days, cartilage-specific collagen Type II was detected, and cartilage-like tissue was formed. This demonstrated that bioprinted HAMA cartilage may find clinical application in repairing cartilage defects.

SLA is well-suited for fabricating microchannel structures, which is challenging to accomplish with extrusion bioprinting. Microchannels play a crucial role in facilitating the efficient transportation of oxygen and nutrients, therefore enhancing cell viability, migration, and proliferation [61]. Melhem et al. [61] utilized SLA to print a cardiac repair patch incorporated microchannels of controlled diameters (500 and 1000 μm) (Figure 4F,G). A bio-ink consisting of a PEGDA solution suspended with mesenchymal stem cells (MSCs) was employed. Ladened with MSCs, the cardiac repair patch consistently released a variety of therapeutic cytokines and exosomes, promoting the effective repair of injured cardiac muscle. Additionally, using a patch with an optimized channel diameter proved to be effective in the reduction of MSC cell loss. In vivo, murine myocardial infarction models were employed to evaluate the efficiency of MSC-laden gel patches containing microchannels. After 8 weeks, untreated mice and those with patches lacking microchannels exhibited left ventricular dilation and wall thinning. In contrast, mice hearts treated with a micro-channeled patch loaded with a higher density of MSCs demonstrated minimal necrosis at the injury site. These treated hearts experienced significantly reduced or negligible ventricular dilation and wall thinning (Figure 4H).

### 2.5. Digital Light Processing, DLP

**Figure 5:**
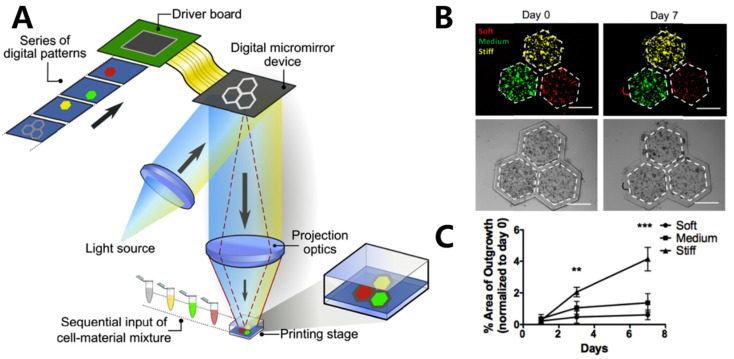
(**A**) Schematic illustration of the bioprinting of hexagonal cell-laden hydrogel scaffolds [67]; (**B**) merged fluorescence images (top) and bright-field images (down) Displaying the recorded positions of HepG2 cells in location to the hexagonal areas over 7 days. Red: soft, green: medium, yellow: stiff condition, scale bar: 500 μm [67]; (**C**) graphical representation illustrating the changing percentage of cell invasion over a period of time, categorized by the three different scaffolds ** *p* ≤ 0.01, *** *p* ≤ 0.001 [67].

DLP is a 3D-printing method that employs a projector based on the digital micromirror device (DMD) or liquid crystal display (LCD) to solidify photoactive bio-inks layer by layer in a pre-designed form (Figure 1B) [32,62]. The fabrication principle endows DLP with several advantages, including high printing speed, excellent accuracy, and improved cell viability. Unlike SLA, DLP utilizes an area light source instead of a laser point, leading to significantly faster printing speeds. Furthermore, DLP employs LED or LCD as a cost-effective alternative to a laser light source. These features position DLP as a highly competitive tissue fabrication method when compared to other printing methods [63,64,65]. Ma and coworkers [66] fabricated a hexagonal GelMA/MeHA hydrogel hepatic model with a stiffness similar to the liver using DLP technology. The model incorporated human-induced pluripotent stem cells (hiPSCs) in conjunction with support human umbilical vein endothelial cells (HUVECs) and adipose-derived stem cells (ADSCs). After 7 days of culture, a more pronounced development of spheroids was observed compared to the model consisting solely of hiPSC-HPCs in the tri-culture 3D model (Figure 5B). In another study, Ma et al. [67] utilized DLP technology to fabricate an in vitro liver model with customizable mechanical properties, serving as a platform to investigate the growth and invasion of hepatocellular carcinoma (HCC) (Figure 5A). The bio-ink, comprising GelMA and liver dECM, employed LAP as the PI. By adjusting the irradiation time (10 s, 20 s, and 40 s), they successfully achieved hexagonal hydrogels with stiffness levels of approximately 0.5 kPa, 5 kPa, and 15 kPa, respectively. These stiffness levels corresponded to different stages of liver cirrhosis (Figure 5C). After 7 days, an increased number of HepG2 cells were observed in the rigid hexagonal scaffold, while fewer HepG2 cells with lower stiffness were observed in the scaffolds (Figure 5C). These results demonstrated the significant potential of the liver model platform for pathophysiological learning and drug screening.

### 2.6. Computed Axial Lithography (CAL)

Although DLP is known for its fast printing speed and high printing resolution, it remains constrained by a two-dimensional accumulation process when constructing 3D structures. This manufacturing approach highlights a constraint in enhancing the printing speed of DLP. To address this issue, Taylor’s team drew inspiration from computed tomography (CT) imaging and developed a layerless technique termed computed axial lithography (CAL), or volumetric printing. This technique facilitates the single-step fabrication of complex 3D structures, as depicted in Figure 1B [32,68]. In this technique, a pre-designed sequence of light patterns is projected onto a printing reservoir containing bio-ink. The reservoir rotates around an axis. The light source simultaneously projects various patterns into the bio-ink. The planar light beam selectively cures the photosensitive bio-ink in the printing reservoir. Consequently, through the accumulation of light exposure, specific regions of the photosensitive bio-ink undergo solidification, enabling volumetric fabrication of 3D objects. The CAL technique successfully addressed the constraints associated with SLA and DLP, particularly their inability to print certain types of bio-inks, especially those characterized by high molecular weight and viscosity. CAL is capable of printing bio-inks with viscosities as high as 9 × 10^4^ cP, therefore effectively broadening the spectrum of printable bio-inks in 3D bioprinting. Furthermore, CAL allows for the volumetric printing of large-sized structures, offering the potential for a significant improvement in printing speed.

Moreover, in the conventional layer-by-layer printing process of traditional SLA and DLP, hydrogel materials with lower moduli are prone to deformation or collapse, and the prolonged printing time can result in cell death within the encapsulated constructs. Furthermore, CAL can also avoid stress caused by layer accumulation, therefore enhancing the viability of encapsulated cells. Thus, CAL demonstrated great potential in the rapid fabrication of intricate hydrogel structures.

## 3. Biological Properties of 3D Bioprinting Hydrogels

In 3D bioprinting, bio-ink refers to a hydrogel-based formulation comprising either a single type of biomaterials or a blend of various biomaterials, along with encapsulated cells. The formulation undergoes further processing by automated biofabrication to form a designed geometry [69,70,71,72]. As light-based bioprinting employs light to fabricate 3D structures, photoreactive moieties must be incorporated into the bio-ink components. Upon exposure to light, a photosensitive compound known as a PI absorbs energy, leading to the generation of reactive species. These reactive species, in turn, trigger the photopolymerization reaction, resulting in the formation of a covalently crosslinked hydrogel [33].

To formulate an ideal bio-ink for light-based 3D bioprinting, meeting the specific mechanical, rheological, chemical, and biological criteria is crucial [7,69]: 1. It should possess biodegradative traits that ensure effective tissue remodeling while preventing any adverse byproducts; 2. When in the presence of cells, it should demonstrate both biocompatibility and minimal immunogenicity; 3. The chosen biomaterial formulation should have tunable mechanical properties to match with different tissues; 4. The printed structures should be able to maintain their structural stability; 5. There should be the possibility of achieving significant production on a large scale while minimizing differences between batches.

Based on these criteria, a growing number of new bio-inks are being developed. However, the scarcity of suitable biomaterials remains a major issue limiting the advancement of tissue engineering. This part discusses the developments in PIs and biomaterials employed for light-based 3D bioprinting.

### 3.1. Photo-Initiators (PIs)

To ensure the successful implementation of 3D bioprinting, the selection of PI is of paramount importance. Given that biofabrication occurs in the presence of living cells, the selected PI should exhibit properties including water solubility, low cytotoxicity, and high extinction coefficient at visible-light wavelength. These characteristics are essential to facilitate fast and high-quality 3D printing of desired structures.

**Figure 6:**
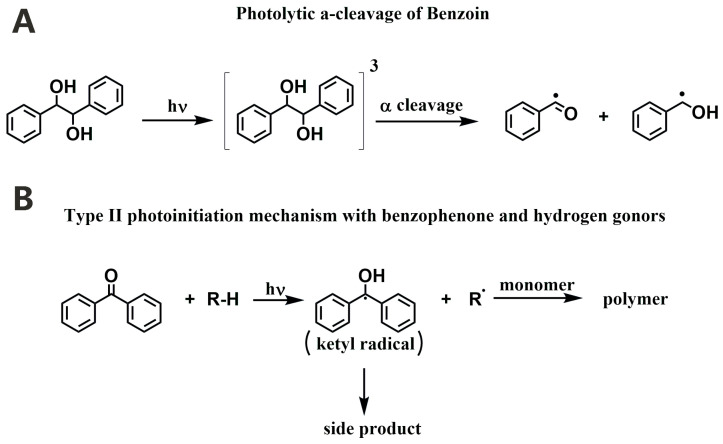
Schematic illustrations of (**A**) cleavage of Type I PI and (**B**) Type II PI interact with hydrogen donor after irradiation [35].

So far, a lot of PIs or PI systems working under visible light have been developed for light-based 3D bioprinting. According to the radical generation mechanism, free radical PIs can be classified into Norrish Type I PI and Norrish Type II PI. Most Type I PIs are aromatic carbonyl compounds, such as benzoin and its derivatives [73], acetophenones [74], phosphinoxides [75,76], and so on. By absorbing photons, they undergo homolytic cleavage and generate two free radicals to initiate the photopolymerization of monomers (Figure 6A) [35]. The initiation of Type II PIs is based on a bimolecular reaction, as illustrated in Figure 6B [35]. The excited Type II PI, benzophenone, in this case, abstracts hydrogen from the hydrogen donor, leading to the generation of initiating radicals. Commonly used Type II PIs include thioxanthone and its derivatives [77,78], camphorquinone (CQ) [79], and so on. In addition, new PIs, including dyes and Ru^2+^ complexes, also have been used as PIs for bio-ink formulations.

#### 3.1.1. Norrish Type I PIs

2-hydroxy-1-[4-(2-hydroxyethoxy) phenyl]-2-methyl-1-propanone (Irgacure 2959, Table 1) is the first and most used commercial water-soluble PI, which has been widely used to fabricate hydrogel networks using materials such as acrylated gelatin (GelMA) [80,81,82], methacrylated chitosan (MeGC, Figure S5) [83,84], and PEGDA [85]. However, the low water solubility and limited absorption in the visible light range of Irgacure 2959 have limited its applications. Although another α-hydroxyketone PI—2-hydroxy-1-[3-(hydroxymethyl)-phenyl)]-2-methyl-1-propanone (APi-180, Table 1)—exhibits enhanced water solubility, it still needs UV light as light sources [86]. Therefore, visible-light PIs should be developed.

In recent years, numerous commercial 3D printers have adopted 405 nm LED light as their light source, making PIs with an absorption range that overlaps 405 nm highly desirable. Monoacylphosphineoxide (MAPO) and bisacylphosphineoxide (BAPO) salts, including LAP, Na-TPO, BAPO-OLi, and BAPO-Ona, are highly reactive and biocompatible visible-light PIs with absorption rings from 380 nm to 450 nm. Significantly, these PIs also boast excellent water solubility and much lower toxicity than Irgacure 2959 (Table 2) [86]. Lunwar et al. [87] developed a tough and compatible double network (DN) hydrogel using DLP. The solution of alginate, dimethyl acrylamide (DMAAm, Figure S6), and methylene bis-acrylamide (MBAAm, Figure S7) were used as monomers, while LAP was used as PI. Results showed that LAP played an important role in the stretchability and stiffness of the DN gel. By optimizing LAP concentration, they found that at a concentration of 0.33 wt.% LAP, the double network (DN) hydrogel exhibited an ultimate stress of 65 ± 4 kPa and an elastic modulus of approximately 50 kPa. Ghazali et al. [88] reported water-soluble TPO nanoparticles with significant absorption in the range from 385 to 420 nm. Furthermore, this TPO nanoparticle could also maintain the high molar excitation coefficient of TPO. This approach to synthesizing PI-based nanoparticles holds promise for the extension to other poorly water-soluble PIs.

**Table 1:** Technologies of light-based bioprinting.

Light-Based Printing Techs	Explanations	Advantages	Disadvantages	Ref.
Inkjet-based bioprinting	Ink droplets are propelled out of a microscopic orifice via thermal or piezoelectric actuation and deposited drop by drop on the platform to fabricate a 3D structure.	High printing resolution. Able to print multi-materials.	Difficult to print large-scale structures. Unable to print with bio-inks of high viscosity. Tend to generate satellite droplets during printing. Shear stress that may impact cell viability.	[41,42,43]
Extrusion-based bioprinting	Selectively deposit bio-inks layer by layer on the printing platform.	Wide range of bio-ink viscosity. Moderate printing time. Able to print multi-materials.	Shear stress that may impact cell viability. Limited printing resolution. Limited complexity of the printed structures. Limited printing speed.	[32,53,54]
Suspension-based bioprinting	Bio-ink is extruded into a gel bath that is immiscible with the printed ink layer by layer, providing adequate support for shaping the bio-ink. After the completion of printing, the gel is washed away.	Provide support for bio-ink with poor mechanical properties. Provide biological environment which supports cell growth. Able to print omnidirectionally. Able to print complex structures with a high aspect ratio.	Limited suspension medium choices.	[48,55,89,90]
SLA-based bioprinting	Focused laser is used to selectively solidify the bio-ink layer by layer.	High printing resolution. Able to manufacture complex structures.	Limited in manufacturing scalable products. Unable to print multi-materials. Only suitable for bio-ink with low viscosity.	[58,59]
DLP-based bioprinting	A projector based on the digital micromirror device (DMD) or liquid crystal display (LCD) is used to solidify photoactive bio-inks with pre-designed form layer by layer.	High printing resolution. High printing speed. Able to manufacture complex structures. Able to manufacture scalable products.	Limited bio-ink choices. Unable to print multi-materials. Only suitable for bio-ink with low viscosity.	[63,64,66]
CAL-based bioprinting	A designed sequence of light patterns is projected onto a rotating printing reservoir containing bio-ink. The bio-ink can be solidified volumetrically.	Able to manufacture complex structures. Rapid printing speed for large constructs. Exceptional fidelity. Smooth surface for the printing structures. Wide range of bio-ink viscosity.	Limited bio-ink choices. Only suitable for transparent bio-inks. Limited printing resolution.	[68,91]

#### 3.1.2. Norrish Type II PIs

CQ and its derivatives are a group of important biocompatible Type II PIs [92]. The addition of an amine co-initiator makes these initiation systems widely applicable in dental restorative materials due to its absorption in the 400–500 nm spectral region [93]. Kowsari et al. [94] reported that an advanced visible-light 3D printing platform employed an organic light-emitting diode (OLED) array with peak emission wavelength at 460, 525, and 625 nm as a light source. Two photoinitiation systems, Irgacure 784 and a combination of CQ/ethyl 4-dimethylaminobenzoate (CQ/EDAB), were used as PIs. This platform enabled the large-scale printing of PEGDA and other bioactive materials with complex structures and high resolution. Furthermore, they successfully achieved multimaterial printing. The results highlight the significant potential of integrating OLED technology and versatile photoinitiation systems for complex, scalable, and multimaterial 3D printing. However, the application of CQ in light-based 3D bioprinting is severely limited by its poor solubility in water (Table 3) [95]. To increase its water solubility, carboxylated CQ with improved solubility was synthesized without changing much of its spectroscopic properties [95,96].

In addition to conventional Type II PIs, dyes like Eosin Y [97,98], riboflavin [99], and Rosa Bengal [100] have recently found applications for light-based 3D bioprinting [101]. When employed as PIs, the addition of an amine electron donor is essential to enhance the photoinitiation efficiency. Eosin Y, a xanthene dye typically employed as a histological stain, exhibits excitation under green light when paired with triethanolamine (TEA) (Tabel 2) [95,102,103]. Fouassier et al. [104] pioneered the innovative application of the Eosin Y/amine photoinitiation system for the fabrication of polyethylene glycol (PEG) base hydrogels. However, Eosin Y tends to suffer from oxygen inhibition, which may hinder photopolymerization. To counter this problem, vinylpyrrolidone (NVP, Figure S8) has been introduced as a comonomer to mitigate this issue and enhance the ultimate conversion of double bonds [105,106]. Aguirre-Soto et al. [105] explored the role of NVP in the co-polymerization with PEGDA in an aqueous environment, employing Eosin Y as the PI. The results indicated that the inclusion of NVP led to reduced oxygen inhibition, increased initial polymerization rate, and enhanced ultimate double-bond conversion. The formation of a ground-state complex between NVP and Eosin may contribute to the reduction of oxygen inhibition, slightly accelerating the speed of photoinduced electron transfer to TEA and resulting in the consumption of two oxygen molecules throughout the process.

Riboflavin, commonly known as vitamin B, stands out as another Type II PI characterized by excellent water solubility and biocompatibility (Table 2) [106,107]. The riboflavin/TEA photoinitiation system displays significant absorption in the wavelength range of 300 to 500 nm [108]. Rosa Bengal has also gained attention as a promising Type II PI, manifesting absorption at 565 nm [109,110]. Ahn et al. [111] introduced a rapid, visible-light tri-component photoinitiation system which was comprised of 5,7-diiodo-3-butoxy-6-fluorone (H-Nu470), Rosa Bengal, and ZnTPP. By employing dimethyl acrylamide and trimethylolpropane triacrylate as monomers alongside this PI system, a DLP 3D printer equipped with exchangeable LEDs achieved rapid printing speeds ranging from 33 to 45 mm/h with low-intensity violet (405 nm), blue (460 nm), green (525 nm), and red (615 nm) light exposure (∼2–3 mW/cm^2^).

Another noteworthy water-soluble visible-light PI is Tris(2,2′-bipyridyl)dichloro-ruthenium(II), or Ru(bpy)_3_^2+^. This metal complex-derived compound exhibits a pronounced absorption peak at 452 nm, as shown in Table 2 [112]. Sodium persulfate (SPS) can be used as its co-initiator. Upon exposure to visible-light, Ru^2+^ undergoes a transition to its excited state and interacts with SPS through an electron transfer process to generate Ru^3+^ and sulfate radicals. These sulfate radicals subsequently initiate the photopolymerization of monomers [113].

**Table 3:** Commercial Type II PIs for 3D bioprinting.

PI	Structure	λ_max_/nm	Solubility in Water	Ref.
CQ	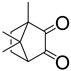	444	slightly soluble in water	[8,95,96]
Eosin Y	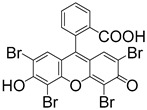	528	300 g/L	[95,104]
Riboflavin	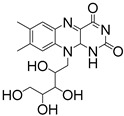	223, 267, 373, 444	100 g/L	[106,107,108]
Rosa Bengal	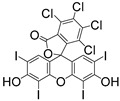	565	100 g/L	[109,110,111]
Ru(bpy)_3_^2+^	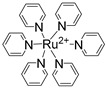	452	excellent solubility in water	[112,113]

### 3.2. Biomaterials for Light-Based 3D Bioprinting

Natural biomaterials, often derived from the proteins and polysaccharides found within living organisms, are favored for bio-inks due to their excellent biocompatibility. Substances like gelatin, chitosan, and alginate, among others, exhibit impressive responsiveness and adhesiveness to living cells. They are also able to undergo degradation within the body. Furthermore, these natural materials are cost-effective and renewable. However, they come with certain drawbacks, including high degradation rates, intricate purification processes, and lower mechanical performance. The most commonly used hydrogel biomaterials, including gelatin, chitosan, alginate, hyaluronic acid, and decellularized extracellular matrix (dECM), will be discussed here.

#### 3.2.1. Gelatin

**Figure 7:**
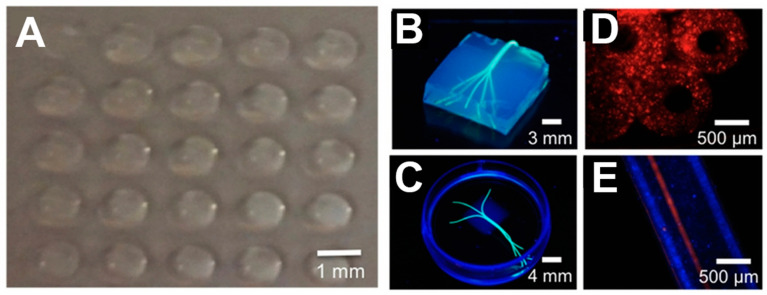
(**A**) Photographs of bioprinted GelMA hydrogel array encapsulated with HepG2 cells [118]; (**B**,**C**) pictures of bioprinted agarose hydrogel fibers imitating 3D branching patterns within GelMA hydrogel blocks [118]; (**D**) fluorescence photo of cells-laden hollow GelMA hydrogel fibers [118]; (**D**) fluorescence photo of cells-laden hollow GelMA hydrogel fibers [118]; (**E**) Image of hollow fibers perfused with a red fluorescent dye by longitudinal view [118].

Gelatin (Figure S9) is derived from denatured collagen and contains an arginine-glycine-aspartic acid (RGD) peptide sequence, which allows for cell attachment and spreading along the hydrogel matrix. Moreover, gelatin incorporates a matrix metalloproteinases (MMPs) peptide, endowing it with the ability to degrade naturally. Importantly, gelatin has already gained FDA approval as a biologically safe material [114]. To introduce photoactivity to gelatin, Van de Bulcke et al. [115] first modified gelatin with methacrylic anhydride (MAA) to obtain methyl acrylated gelatin, commonly known as GelMA. GelMA contains methacrylate and methacrylamide groups, as both hydroxyl groups and amino groups can react with MAA. This modification did not alter the RGD sequence and MMP sequence of gelatin so that GelMA maintains its excellent biocompatibility and enzymatic degradability. Consequently, GelMA has gained significant attention in the field of 3D bioprinting [116,117]. Bertassoni et al. [118] employed direct writing to produce cell-laden GelMA hydrogel constructs using solutions of GelMA with concentrations ranging from 5% to 15% (Figure 7A–C,E). Notably, the GelMA hydrogel with a concentration of 15% exhibited an impressive elastic modulus of 60.3 ± 9.5 kPa. Furthermore, all the printed scaffolds which encapsulated with HepG2 cells exhibited remarkably high cell viability (Figure 7D).

Although GelMA is an excellent material for tissue engineering and is frequently employed in chain-growth photopolymerization, the resulting hydrogel network may exhibit heterogeneity, which can be detrimental to the encapsulated cells. To address this issue, by modifying gelatin with allyl glycidyl ether (AGE) under alkaline conditions at a temperature of 65 °C, Bertlein et al. [119] synthesized a monomer called GelAGE that is suitable for thiol-ene photopolymerization (the synthetic routine is shown in Figure S10). In comparison between the photopolymerization product of the GelMA system and the GelAGE/dithiothreitol (DTT) system, they found that under the same photo-initiator concentration and light exposure, the mechanical properties of the GelAGE/DTT system were superior to those of the GelMA system. Additionally, GelAGE exhibited shear thinning, enabling the preparation of inks suitable for both digital light processing (DLP) and extrusion-based bioprinting by adjusting the ratio of GelAGE and DTT.

#### 3.2.2. Chitosan

Chitosan (Figure S11), derived from the deacetylation of chitin and primarily composed of glucosamine units, serves as the primary structural component found in crustacean exoskeletons and is an assembly constituent of glycosaminoglycan in ECM [12,22]. With features such as biodegradability, biocompatibility, non-toxicity, antibacterial properties, anticancer effects, lipid-lowering capabilities, and immune enhancement, it finds extensive applications in fields like drug delivery [120,121], medical absorbable materials [122], tissue engineering [123], and pharmaceutical development [124]. Its macromolecular chain contains an abundant amount of amino groups, hydroxyl groups, and ether bonds, allowing for modification reactions such as acylation, esterification, carboxylation, etherification, oxidation, and Schiff base formation [125,126]. However, chitosan’s limited solubility in water restricts its use as a bio-ink, as it can only dissolve in acidic solutions. Consequently, the application of chitosan as a bio-ink has been limited [127]. To address this challenge, a water-soluble chitosan derivative called glycol chitosan (GC, Figure S12) is synthesized by the conjugation of ethylene glycol to chitosan [67]. To further impart GC with photoreactivity, methacrylated GC (MeGC, Figure S13) was synthesized via the methacrylation of reactive amine groups of GC. Upon irradiation, MeGC can undergo photopolymerization [128].

#### 3.2.3. Alginate

**Figure 8:**
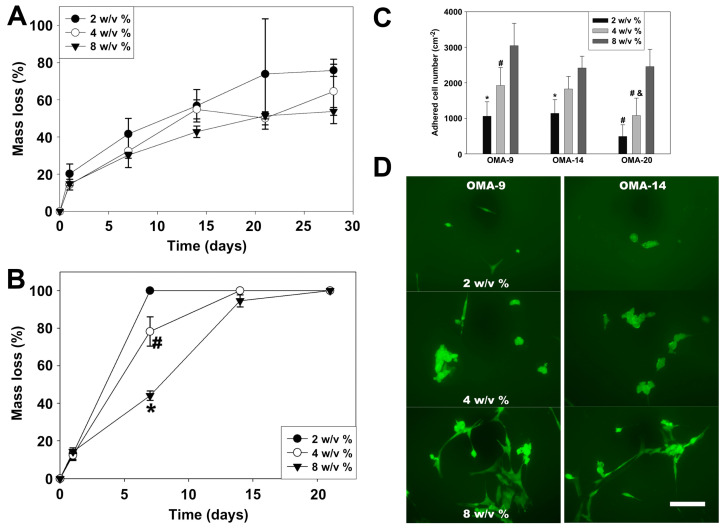
The mass loss of OMA hydrogels (**A**) OMA-9 and (**B**) OMA-14 in DMEM within 30 days [133]; (**C**) the adherent cell number on OMA hydrogels with different oxidation ratio [133]; (**D**) fluorescence images of hBMMSCs cultured on the surface of OMA hydrogels in vitro after 7 days, scale bar: 200 μm [133]. * *p* < 0.05 compared to 2 and 4 *w*/*v* %, # *p* < 0.05 compared to 2 *w*/*v* %, & *p* < 0.05 compared to OMA-9 and OMA-14.

Alginate (Figure S14), a natural polysaccharide polymer extracted from brown seaweed, is characterized by its excellent water solubility, biodegradability, and biocompatibility. It consists of homopolymeric blocks of (1→4)-linked β-D-mannuronate (M) and α-L-guluronate (G) residues covalently linked together in different sequences or blocks [129,130]. As alginate molecules contain numerous carboxyl groups that are negatively charged, hydrogels can be formed through ionic crosslinks with multivalent cations (Ca^2+^, Ba^2+^, Fe^3+^, and so on) [131]. Similar to chitosan and gelation, alginate can also be modified for photopolymerization. Photocrosslinkable methacrylated alginate (Alg-MA, Figure S15) can be synthesized by treating the secondary hydroxyl groups with MAA [132]. Carboxylic groups on the alginate molecular chain are also modification sites. By modifying these carboxylic groups with aminoethyl methacrylates, photocrosslinkable alginate can also be obtained. Jeon et al. [133] synthesized oxidized and methacrylated alginates (OMA, Figure S16), where first, mannuronate groups were oxidized by NaIO_4_, and subsequently, carboxylic groups were activated by EDC/NHS (1-ethyl-3-(3-dimethylaminopropyl)carbodiimide/N-hydroxysuccinimide) and methacrylate groups were introduced using 2-aminoethyl methacrylate (AEMA). Then, a range of photocrosslink biodegradable hydrogels, featuring adjustable swelling ratios, stiffness, and degradation rates were prepared through the utilization of OMA. Results showed that the OMA hydrogel with a 14% oxidation ratio (OMA-14) degrades faster than the OMA with a 9% oxidation ratio (OMA-9) (Figure 8A,B). Moreover, the OMA hydrogels exhibited improved human bone marrow mesenchymal stem cell (hBMMSC) adhesion and spreading, as observed in Figure 8C,D. This phenomenon can be attributed to the rich aldehyde groups with the OMA hydrogels, which can interact with the amines present on cell surface proteins in the extracellular matrix.

Norbornene functionalized alginate (Alg-NB, Figure S17) and phenyl group functionalized alginate (Alg-Ph, Figure S18) were also synthesized [134,135,136]. These monomers can be used in photo-click reactions and photo-mediated redox crosslinking, respectively. Ooi et al. [134] developed a bio-ink composed of Alg-Nb and thiol crosslinker CGGGRGDS peptides (Figure S19). By adjusting thiol crosslinker content in the bio-ink, they successfully fabricated alginate-based scaffolds using extrusion-based 3D bioprinting. The moduli of the obtained hydrogels ranged from 0.05 to 30 kPa, indicating their potential to be used as tissue-engineering scaffolds. After 7 days of culture in vitro, the viability of the mouse fibroblast (L929 cells) encapsulated in the printed hydrogel was above 80%. They further modified Alg-Norb with HS-RGD bioactive peptide through thiol-ene photo-click reactions, enabling alginate-based hydrogel to modulate cellular behavior and induce cell proliferation and differentiation.

#### 3.2.4. Hyaluronic Acid

**Figure 9:**
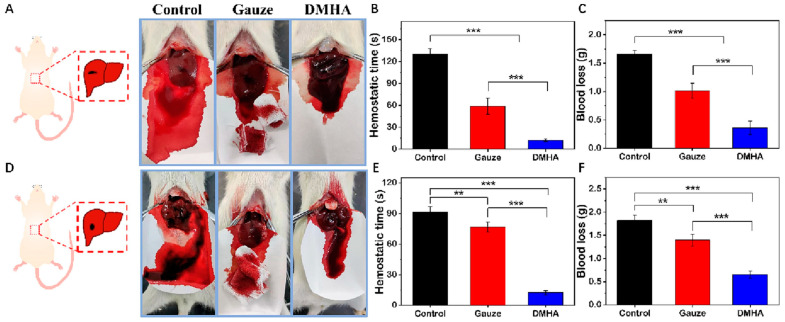
DMHA hydrogels’ hemostatic performance evaluation. The rat liver injury model used DMHA hydrogel for hemostasis (**A**–**C**), and the rat liver noncompressible hemorrhage model did not use DMHA hydrogel (**D**–**F**). Statistical data represent mean ± SD, and ** *p* < 0.01, *** *p* < 0.001 [149].

Hyaluronic acid (HA, Figure S20), also known as hyaluronan, stands as the predominant constituent in ECM of human epithelial, connective, and neural tissues [137]. It is a non-sulfated glycosaminoglycan composed of disaccharide units consisting of N-acetyl-D-glucosamine and β-D-glucuronic acid linked by β-1,3 and β-1,4 glycosidic bonds [138]. Due to its remarkable biocompatibility and biodegradability, HA has found applications in orthopedics and plastic surgery [139]. Its natural biological functions, including binding to the cell surface receptor CD44 [140,141] and susceptibility to degradation by mammalian enzymes [142], made HA a promising material in the field of 3D biofabricaiton [143,144,145,146]. Photoactive groups such as methacrylate, vinyl, and cineole can be introduced to the HA backbone through abundant hydroxyl groups via crosslinking, grafting, and esterification reactions. The most common methods for modifying HA involve the methacrylation of its primary hydroxyl groups through reactions with MAA or glycidyl methacrylate [147,148]. Fan et al. [149] developed a flexible HA hydrogel adhesive for effective hemostasis. They first synthesized an HA derivative called dopamine-conjugated maleic hyaluronic acid (DMHA, Figure S21). This derivative, enriched with acrylate groups and dopamine groups, was achieved by modifying HA with dopamine and MAA. Subsequently, DMHA was coated on rat liver and exposed to UV light. Due to the presence of thiol groups on rat tissue, DMHA formed effective adhesion to the wounded liver. Impressively, DMHA exhibited a short hemostasis time of merely 12.2 ± 1.9 s (Figure 9A–F). However, a high double-bond grafting ratio is not always beneficial for photopolymerization. As the grafting ratio increased, the double-bond conversion initially increased and then decreased. The presence of unreacted double bonds could potentially have a negative impact on the biocompatibility of HA derivatives. To address this issue, Loebel et al. [150] prepared tyramine-functionalized hyaluronic acid (HA-Tyr, Figure S22) and utilized erythrosine Y (EO) and Rosa Bengal (RB) as PIs for the 3D bioprinting of hydrogels encapsulated with mesenchymal stem cells (MSCs). HA-Tyr could gel within only 30 s when the mass fractions of EO and RB were 0.02%. Additionally, HA-Tyr demonstrated relatively low viscosity, making it suitable for lithography-based bioprinting. The authors further printed scaffolds with high precision using laser direct writing and two-photon polymerization, respectively.

#### 3.2.5. Decellularized Extracellular Matrix (dECM)

The extracellular matrix (ECM) in the human body consists of fibrous proteins and glycosaminoglycans (GAGs). It serves not only as a structural framework but also dynamically influences cellular behavior, regulating cell activities, including cell proliferation, cell differentiation, cell migration, and intercellular communication [151]. Moreover, the components of the ECM of each tissue or organ differ in providing suitable microenvironments for specific cell populations [152]. Designing biomaterials and structures with unique properties that closely mimic the targeted tissue is crucial to enhancing the functionality, phenotype, and maturation of encapsulated cells, therefore facilitating tissue regeneration. However, it is quite challenging to fulfill the fabrication of a complex cellular environment, given the current state of biomaterials development and fabrication methods. To synthesize a biomaterial that is completely identical to ECM is not feasible now.

**Figure 10:**
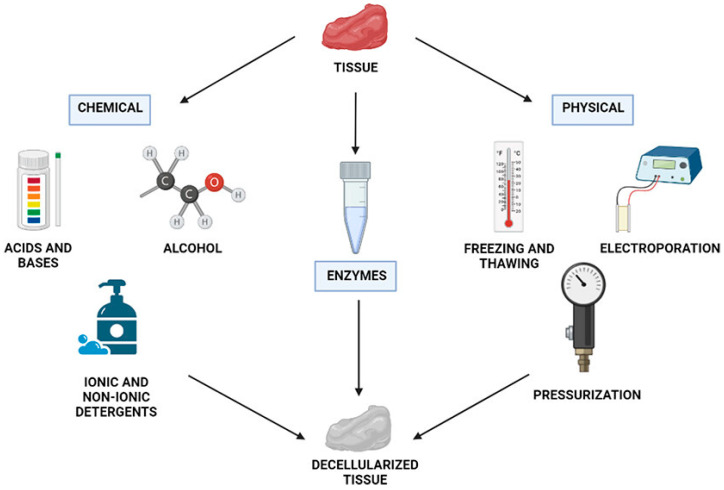
Schematic illustration of decellularization strategies [158].

In 1973, Elliott et al. [153] first reported the dECM technique, in which ECM was extracted from porcine skin and utilized for wound dressing. Later, in 2008, Badylak [154] reported a method for preparing hydrogels using ECM extracted from porcine bladder tissue. When ECM components are extracted from tissues to form dECM, it not only preserves the microenvironment required for cell growth but also demonstrates exceptional biocompatibility. The unique mesh-like architecture of the ECM imparts distinct mechanical properties to various tissues and organs, providing the necessary habitat for cell growth and development. Moreover, the ECM itself does not contain genetic material components, rendering it non-immunogenic and highly biocompatible. Evidence also showed that dECM, when used as a scaffold, can provide responsive biological factors for cell proliferation and differentiation even without adding exogenous growth factors [155,156]. DECM not only exhibits excellent flowability and injectability but also demonstrates gelation ability at 37 °C [157]. This allows dECM to be used as a minimally invasive injectable implant, promoting tissue repair and regeneration. To prepare dECM without cells while retaining its unique tissue consumption, a blend of mechanical disruption, enzymatic breakdown, and chemical cleaning methods can be used (Figure 10) [158]. To date, a variety of protocols have been developed to process different kinds of dECM, such as heart, liver, adipose, lung, and so on [159,160,161]. Thus, extensive research [162] has been done on the dECM, and dECM has been used as scaffolds for various tissues such as skin [163], heart [164], bone [165], nerve [166], liver [167], kidney [168], lung [159], and so on.

To realize fast and precise fabrication of highly complex structures, the preparation of dECM suitable for light-based bioprinting, particularly for lithography-base bioprinting, has become a research hotspot [169].

### 3.3. Properties of Bio-Inks

Bio-inks must possess exceptional physicochemical and biological properties to ensure both printability and biocompatibility. Printability indicates the capability of bio-inks to construct highly intricate 3D structures with precision and fidelity. Biocompatibility indicates the ability of bio-inks to interact with cells, thus promoting cell adhesion, proliferation, and spreading. Balancing the physicochemical and biological properties of bio-inks is crucial for the successful implementation of 3D bioprinting and the realization of biologically functional tissue fabrication [170,171].

3D bioprinting fabricates 3D constructs through layer-by-layer deposition or solidification of bio-inks. First, the bio-ink used should be able to maintain its structural stability after printing while minimizing its impact on the viability and density of encapsulated cells during the printing process. For photocrosslinkable bio-inks, let us take GelMA as an example. The hydrogel network forms through chain-growth photopolymerization of methacrylate double bonds. However, during photopolymerization, formed free radicals are easily trapped within the crosslinked network, resulting in a high local concentration of radicals. Excessive crosslinking can yield a brittle and fragile polymer, while insufficient crosslinking leads to a soft yet tough polymer. Moreover, the formed hydrogel network may exhibit a significant degree of microstructural heterogeneity with unevenly distributed crosslinking regions. This could result in a significant degree of microstructural heterogeneity in the hydrogel network [172]. These factors may result in the deformity or mechanical breakdown of the construct, further influencing the fate of the encapsulated cells. Ensuring an appropriate degree of crosslinking is crucial for the polymer network structure. Thiol-ene photoreaction is quite effective in building highly uniform hydrogel networks with reduced shrinkage and mechanical stress [173,174]. This photo-click reaction exhibits orthogonal behavior, meaning that one thiol group only reacts with one double bond, leading to the formation of a homogeneous hydrogel network [47].

The rheological property is also of crucial importance for bioprinting. For extrusion printing, a bio-ink of high viscosity can better maintain the printed structure and enhance mechanical stability. However, high-viscosity bio-ink may lead to nozzle clogging and stress forces on the encapsulated cells. On the contrary, bio-ink with low viscosity does reduce the shear forces and is less likely to clog the nozzles but struggles to maintain the structural integrity of the printed structures. The use of bio-ink with shear-thinning properties is highly needed to solve these problems. GelMA and alginate are typical biopolymers that show shear-thinning properties [175,176]. With the emergence of shear-thinning bio-inks, extrusion through micronozzles has become possible and has led to an improvement in bioprinting resolution. In lithography-based bioprinting, bio-ink viscosity also plays a significant role. In contrast to the bio-inks used in extrusion bioprinting, the viscosity of bio-ink for lithography-based bioprinting should be low enough to maintain fluidity to ensure successful printing [170].

Bio-inks should also possess biocompatibility, controllable degradability, and angiogenic capability. Cell-laden bio-inks are needed to maintain high cellular viability. As a result, biomaterials with properties like non-toxicity, non-allergenicity, and non-irritation are employed as bio-inks. Consequently, natural biomaterials, which offer better biocompatibility and improved interactions with cells, are extensively utilized in the field of bioprinting. Numerous bio-inks based on natural biomaterials with excellent biocompatibility have been developed [71,177,178].

In summary, the physicochemical and biological properties of bio-inks are of crucial importance for the construction of bioprinted structures. However, achieving excellent physicochemical and biological properties simultaneously proves challenging. For instance, higher ink concentrations are often necessary to maintain mechanical strength and printability, leading to a higher crosslink ratio and subsequently lowering the porosity and pore sizes of a printed structure. However, a sparse crosslinked network that facilitates nutrient and oxygen exchange is also essential for cell encapsulation. Achieving a balance between these factors is challenging. Hence, the future direction of bio-ink development lies in creating bio-inks with both good physicochemical properties and biological properties.

## 4. Applications of Light-Based Bioprinting

The tissues and organs of the body possess complex hierarchical structures, where the 3D microstructure and microenvironment play pivotal roles in promoting cell viability and guiding various cellular activities such as cell adhesion, proliferation, and migration. Nevertheless, replicating tissue structures that closely resemble the ECM of human tissue is quite challenging due to limitations in manufacturing technology. The emergence of 3D bioprinting, especially light-based bioprinting, has made this possible. The advent of light-based bioprinting has significantly advanced the ability to fabricate intricate microstructures, making it possible to fabricate complex tissue structures such as liver [179], skin [180], bone and cartilage [181], and cardiac tissues (Table 4) [182]. The applications of light-based bioprinting are discussed in this section.

### 4.1. Liver Tissue Engineering

**Figure 11:**
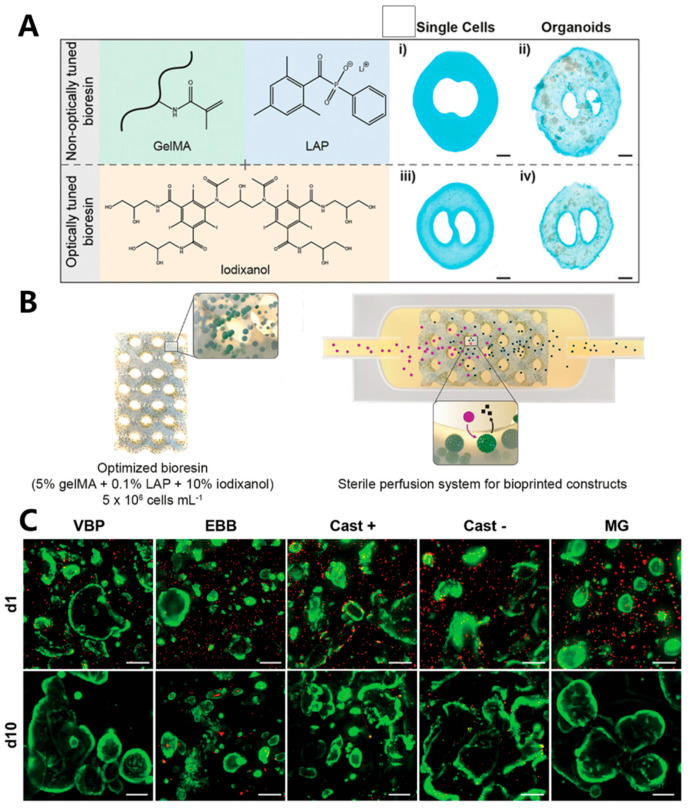
(**A**) Schematic illustration of the photoactive bio-ink employed for volumetric bioprinting composed of GelMA and LAP, where iodixanol was used as supplementation to optically adjust the bio-ink for improved printing precision with the presence of organoid. (i, ii) stereomicroscopy images displaying non-optically tuned bioresin, and (iii, iv) images displaying iodixanol-containing bioresins designed for the 3D printing of single cells and organoids. The scale bars represent 1 mm [179]; (**B**) Schematic representation of a complex, organoid-laden bioprinted biofactory cultivated in dynamic perfusion [179]; (**C**) Live/dead cell photos over 10 days cultivation, scale bars: 250 µm [179].

The liver, the largest gland in the human body, plays a vital role in various physiological processes, including metabolism, bile production, detoxification, and the regulation of water and electrolyte balance. Liver diseases have become prominent causes of mortality worldwide over the past few decades. Therefore, there is an urgent need to develop liver scaffolds and in vitro liver models for purposes such as liver regeneration, drug screening, metabolism research, and the study of hepatotoxicity [187]. Light-based bioprinting has been employed in the biofabrication of liver tissue [183]. Bernal et al. [91] fabricated a GelMA hydrogel scaffold containing articular cartilage progenitor cells (ACPCs) using CAL. They successfully printed complex cartilage-like scaffolds and trabecular bone scaffolds in just a few seconds, surpassing precision extrusion-based and DLP-based bioprinting. After 28 days of in vitro cultivation, the printed meniscus-like structures exhibited significantly increased ACPC metabolic activity. These scaffolds demonstrated uniform distributions of glycosaminoglycans (GAGs) and Type I collagen with high content, while Type II collagen content was lower, indicating that CAL-printed structures provide optimal conditions for cell attachment, migration, and proliferation. Bernal et al. [179] extended the application of CAL to fabricate liver-like metabolic biofactories. Using GelMA as bio-ink and iodixanol as supplementation, a hepatic organoid with a microscale multicellular structure was created. Using iodixanol, positive and negative channel structures of high resolution were fabricated (41.5 ± 2.9 µm and 104.0 ± 5.5 µm, respectively) (Figure 11A,B). The authors compared the cell viability encapsulated in CAL-fabricated hydrogel and extrusion-fabricated hydrogel 1 day after printing. Live/dead fluorescent staining indicated organoids printed via CAL showed excellent viability (93.3 ± 1.4%) and undisturbed average size (273.5 ± 49.9 µm) compared to that of traditional extrusion printing (73.2 ± 1.2% viability, 100.1 ± 14.2 µm average size) (Figure 11C). The results suggest potential applications in the field of tissue engineering and regenerative medicine. Mao et al. [207] developed a liver-specific bio-ink composed of GelMA and porcine liver dECM (GelMA/dECM), along with human-induced hepatocytes (hiHep cells) encapsulated. Utilizing DLP, they printed liver-like microtissue, which demonstrated superior printability and higher hiHep cell viability compared to GelMA bio-ink. Jang et al. [208] developed a two-step process that utilizes sequential vitamin B2-induced UVA crosslinking and thermal gelation to solidify the dECM bio-ink. Initially, the vitamin B2-mixed dECM bio-ink, containing encapsulated cardiac progenitor cells (CPCs), was drawn into a syringe. The syringe was equipped with a low-temperature controller to ensure that the bio-ink maintains its fluidity during extrusion. UVA exposure is applied to initiate the photocrosslinking of heart dECM bio-ink after the extrusion of each layer. The resulting structure was subsequently incubated in a 37 °C environment, allowing the dECM to undergo gelation to provide additional mechanical strength to the printed tissue structure.

### 4.2. Cardiovascular Tissue Engineering

**Figure 12:**
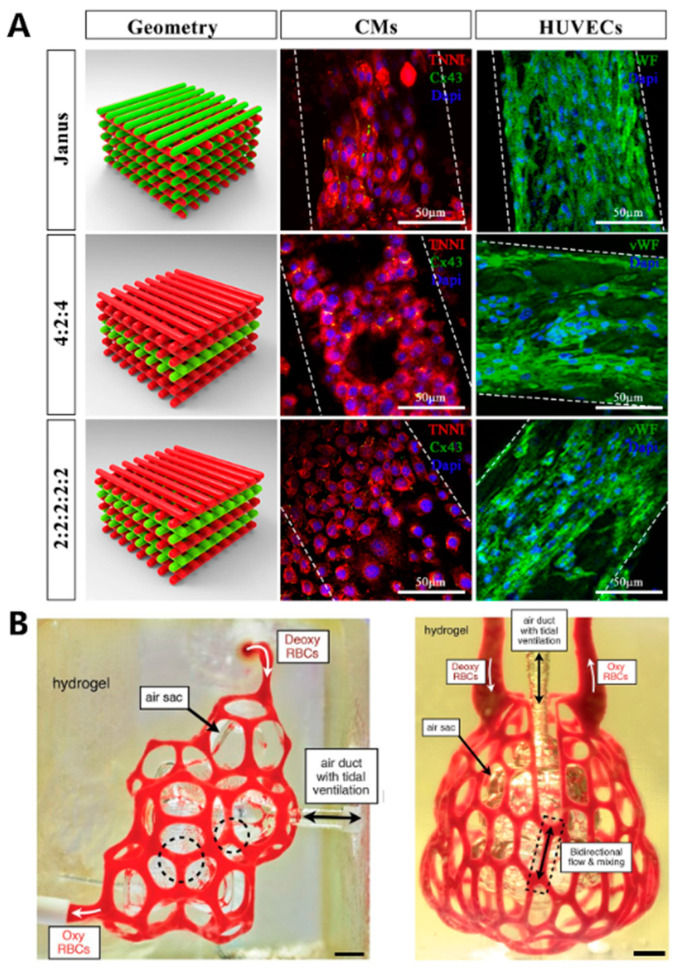
(**A**) Cardiomyocytes and HUVECs were printed in 3 different spatial geometries within a single strand (Janus) or in alternating layers with multi-materials after 7 days of cultivation. Rabbit monoclonal antibody against anti-cardiac troponin I (TNNI, red) and connexin 43 (Cx43, green) expressions in CMs and sheep anti-von Willebrand (vWF, green) labeling in HUVEC. Scale bars: 50 μm [197]. (**B**) The printed lung structure containing intricate and functional vascular architectures. Scale bar: 3 mm for the left image and 1 mm for the right image [40].

Cardiovascular diseases are a category of illnesses that affect the heart muscle, heart valves, or the pathology of blood vessels within the body [209]. In recent years, the incidence of cardiovascular diseases has been steadily rising, posing a substantial threat to human life and health. Conventional treatments, including medications, surgery, and interventional procedures, can only provide relief from clinical symptoms but do not fundamentally address the issue. In the field of regenerative medicine, 3D printing technology represents a significant opportunity, as 3D-printed implantable organs hold the potential to contribute to saving more lives [210]. Maiullari et al. [197] employed a bio-ink composed of PEG-fibrinogen (PEG-PF) and sodium alginate (PEG-PF/Alg), along with human umbilical vein endothelial cells (HUVECs) and patient-specific induced pluripotent stem cells (iPSC-CMs) encapsulated within the bio-ink. They employed a customized microfluidic printing head on an extrusion 3D bioprinter to print high-fidelity heart and vascular structures (Figure 12A). The porous structure of the 3D printed hydrogel facilitated significantly enhanced cell proliferation encapsulated within the printed structure compared to the bulk gel. After in vitro cultivation, a notable increase of α-myosin heavy chain (MHC) expression in the printed heart structure suggested the growth and proliferation of cardiac cells. Results from in vivo transplantation demonstrate that the printed cardiac tissue can integrate with the host vascular system, enabling the regeneration of vascular tissue. In 2019, Grigoryan et al. [40] utilized DLP printing to fabricate PEGDA hydrogel containing intricate and functional vascular architectures, where food dyes with visible-light wavelengths were used as PIs (Figure 12B). The resulting vascular architectures exhibited the capability for oxygen exchange.

### 4.3. Skin Tissue Regeneration

The skin, the body’s largest organ, consists of three distinct layers: the epidermis, the dermis, and subcutaneous tissue [211]. Light-based bioprinting opens up possibilities for the fabrication of scalable skin structures. Based on bioengineering, Borris et al. [199] created a 3D skin model that mimics the complex, multilayered structure of natural skin. This model integrates layers of endothelial cell networks, dermal fibroblasts, and multiple layers of keratinocytes. Their investigation of the mechanical properties of GelMA-based bioresins blended with varying ratios of alginate revealed that the bioprinted endothelial layer could be more effectively simulated to enhance endothelial cell viability when using a combination consisting of 7.5% GelMA and 2% alginate. They also observed that the stiffness of the hydrogel played a crucial role in regulating the expression of pro-collagen I alpha-1 and matrix metalloproteinase-1 in human dermal fibroblasts. Additionally, the repeated gelatin-coating of human keratinocytes proved beneficial in reducing culture duration while maintaining their viability, enabling the creation of multiple layers of keratinocytes.

### 4.4. Bone Tissue Regeneration

**Figure 13:**
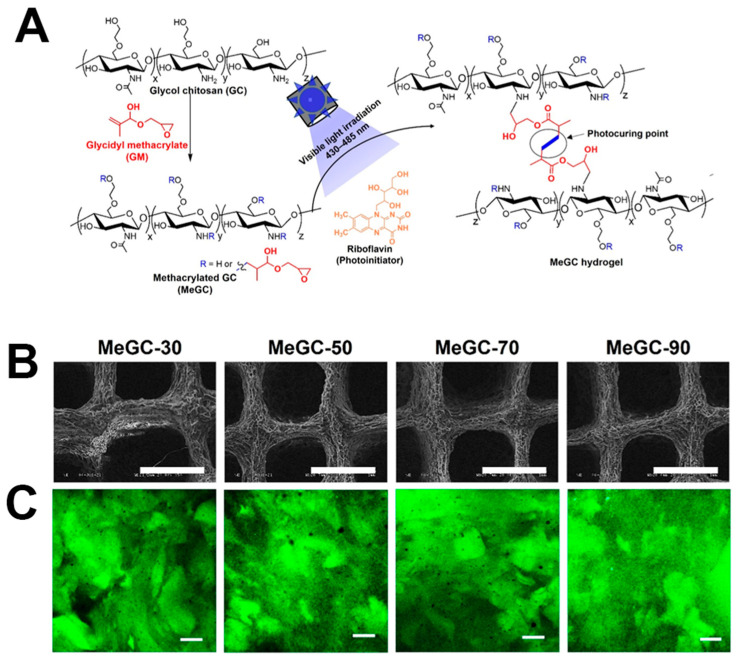
(**A**) Diagram depicting the process of MeGC hydrogel preparation [216]; (**B**) SEM images 3D printed MeGC-30, MeGC-50, MeGC-70, and MeGC-90 scaffolds prepared by freeze-drying of hydrogels, scale bar: 1 mm [216]; (**C**) Confocal microscopic photos of the hydrated scaffolds, scale bar: 200 μm [216].

Bone, a vital tissue in the human body, provides essential support and protection to our organs. However, the natural self-repair capacity of human bone tissue is limited, especially when addressing significant bone defects. Currently, the primary method for repairing and reconstructing damaged bone tissue involves bone transplantation surgery. Nevertheless, this approach faces significant challenges, including a shortage of available donors and the risk of immune rejection. In response to these challenges, bone tissue engineering has emerged as a promising alternative solution [212]. An ideal bone tissue-engineering scaffold should encompass several essential characteristics: (1) Mechanical mimicry: It should mimic the mechanical properties of natural bones to ensure sufficient support within the human body; (2) Osteoconductive and osteoinductive properties: The scaffold should promote the differentiation of cells into osteogenic cells, creating an environment conducive to bone regeneration; and (3) Seamless vascular integration: It should be capable of seamlessly integrating with blood vessels, facilitating the transport of oxygen and nutrients [213,214]. The utilization of light-based bioprinting techniques achieves an exceptional degree of uniformity in the distribution of bone-related cells within the printed structures [171,215]. This precision in cell placement not only reduces the likelihood of necrotic areas forming but also enables the smooth integration of blood vessel structures into the engineered tissues. Chang et al. [216] utilized a MeGC-based bio-ink laden with human osteosarcoma cells MG-63 for light-based extrusion printing, employing riboflavin as PI (Figure 3A). The cell-laden MeGC bio-ink exhibited favorable printability, resulting in 3D constructs with excellent shape fidelity (Figure 13A,B). Even after 7 days, the viability of MG-63 cells remained high. Furthermore, following 7 days of cultivation in an osteoinductive media, the MG-63 cell-laden constructs demonstrated enhanced osteogenic differentiation. Throughout this period, the ALP activities of MG-63 cells within MeGC bio-inks displayed consistent increments. This scaffold shows promise for bone tissue repair. Rajabi et al. [191] conducted a study where they developed bone scaffolds using a variety of photocrosslinkable bio-inks composed of chito-oligosaccharide (COS) and PEGDA for extrusion-based bioprinting. The printed scaffolds were crosslinked using the aza-Michael addition of COS and PEGDA. Additionally, the unpolymerized PEGDA was further crosslinked through exposure to UV light. During the extrusion process, the bio-ink exhibited shear-thinning properties, which are beneficial for smooth and consistent printing. The bio-ink also demonstrated excellent fidelity and high printing resolution. One noteworthy finding was that the swelling ratio of the bone scaffolds decreased as the ratio of COS in the bio-ink increased. Furthermore, the study found that when the weight ratio of COS in the bio-ink was 2%, the resulting bone scaffolds displayed significant improvements in alkaline phosphatase activity, calcium deposition, and bioactivity when compared to pure 3D PEGDA bone scaffolds. This indicates that the inclusion of COS in the bio-ink formulation had a positive impact on the bioactivity and functionality of the fabricated bone scaffolds.

## 5. Summary and Outlook

By assembling biomaterials, cells, and growth factors on demand, 3D bioprinting has evolved into an advanced manufacturing method for building extremely intricate constructs for tissue engineering and regenerative medicine. Light-based 3D bioprinting has become an even more powerful tool in the fabrication of highly complex structures due to its incomparable spatiotemporal control over the chemical, physical, and biological properties of photoactive biomaterials. So far, extrusion bioprinting, inkjet bioprinting, and lithography bioprinting are actively utilized in the field of tissue engineering. Extrusion and inkjet bioprinting are widely used in this field because of their advantages, such as cost-effectiveness, ease of use, perfect adaptation for supporting living cells, and a wide range of suitable biomaterials. Moreover, lithography continues to gain more attention due to its rapid printing speed, high printing resolution, and friendliness to encapsulated cells. In addition to light-based bioprinting methods, extensive research has also been conducted on bio-inks. The photoactivity and biological properties of bio-inks are critically important for 3D tissue structure bioprinting. A bio-ink formulation is composed of PI, monomers, encapsulated cells, and other additives. Type I photo-initiators (PIs), such as Irgacure 2959, MAPO, and BAPO derivatives, as well as Type II PIs, including Eosin Y, riboflavin, Rosa Bengal, and Ru(bpy)_3_^2+^, are commonly used in bio-ink formulations. Natural biomaterials, like gelatin, chitosan, alginate, hyaluronic acid, and decellularized extracellular matrix (dECM), are frequently employed in bio-ink formulations due to their excellent biocompatibility and rheological properties. To make them suitable for photocrosslinking, these materials are often modified with acrylate groups, thiol groups, norbene groups, and so on. With the assistance of 3D bioprinters and suitable bio-inks, 3D printed structures have already found applications as tissue substitutes and tissue models for tissue regeneration, drug testing, and the study of pathophysiology for organs. Thus, this article reviews the latest advancements in light-based bioprinting. Light-based bioprinting techniques, including inkjet bioprinting, extrusion bioprinting, SLA, DLP, and CAL, have been introduced. Then, bio-ink formulations composed of natural biomaterials and commonly used PIs are introduced. The physicochemical and biological properties are discussed. Moreover, the applications of light-based bioprinting in the fields of liver tissue engineering, cardiovascular tissue engineering, skin tissue engineering, and bone tissue engineering were discussed.

However, current light-based 3D bioprinting also faces some challenges.

1. The printing methods have limited printing resolution, up to 20 μm. Thus, the development of novel printers capable of achieving higher printing resolution is quite necessary.
2. Although many biomaterials and PIs have been developed recently, the variety of materials for light-based extrusion 3D bioprinting is relatively limited. The selection of bio-inks suitable for SLA and DLP remains constrained. Hence, the development of new bio-inks and a universal bio-ink toolbox for 3D bioprinting is an important direction.
3. The production of cell-damaging species, such as initiator fragments, has a negative impact on cell fate and viability. Developing efficient and non-toxic PIs is, therefore, essential. An effective strategy for enhancing the biocompatibility of bio-ink formulations is to develop macromolecular photo-initiators with low mobility.

## Figures and Tables

**Table 2:** Commercial Type I PIs for 3D bioprinting.

PI	Structure	λ_max_/nm	Solubility g/L	Toxicity LC_50_ [mmol/dm^3^]	Ref.
Irgacure 2959	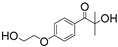	296	5	9.0	[86]
APi-180	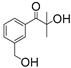	329	74	8.7	[86]
LAP	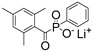	375	47	3.1	[86]
Na-TPO	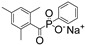	380	29	˂0.56	[86]
BAPO-OLi	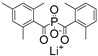	383	54	2.6	[86]
BAPO-ONa	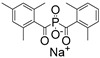	383	60	2.8	[86]

**Table 4:** Applications of light-based bioprinting in tissue engineering.

Tissue or Organ Type	Light-Based Bioprinting Technology	Applications	Development Directions	Ref.
Liver	Extrusion	Fabrication of biological livers and in vitro liver models for purposes such as liver regeneration, drug screening, metabolism research, and the study of hepatotoxicity	The construction of a perfusable high-density biomimetic vascular network in the printed liver tissue.	[66,179,183,184,185,186,187]
	SLA			
	DLP			
	CAL			
Bone	Inkjet	Fabrication of a framework to improve cell adhesion, proliferation, and differentiation, then to further integrate with the native tissue.	Developing bio-inks with improved mechanical properties for seamless integration with native bone tissue.	[188,189,190,191,192,193,194]
	Extrusion			
	SLA			
	DLP			
	CAL			
Cardiac tissue	Extrusion	Fabrication of cardiac tissue capable of the regeneration of different structures in a human heart. Building in vitro cardiac models for studying cardiovascular diseases	To develop materials with good flexibility and elasticity suitable for the encapsulation of related cells.	[39,40,61,195,196,197,198]
	Suspension			
	SLA			
	DLP			
Skin	Inkjet	Printing skin replicas for the repair of skin damage. Fabricating skin models to study the pathophysiology of skin diseases.	Developing skin-specific biomimetic bio-inks and the regeneration of skin appendages. Developing multilayered complex skin models.	[180,199,200,201]
	Extrusion			
	SLA			
	DLP			
Cartilage	Inkjet	Printing cartilage implants for the regeneration of damaged cartilage. Building models for drug testing	Fabrication cartilage structures exhibit mechanical compatibility with the damaged sites. Constructing integrated bone-cartilage tissues and grafts	[60,181,202,203,204,205,206]
	Extrusion			
	SLA			
	DLP			
	CAL			

## Data Availability

Not applicable.

## Supplementary Materials

The following supporting information can be downloaded at: https://www.mdpi.com/article/10.3390/ma16237461/s1. Figure S1. Structure of HAMA. Figure S2. Structures of maleiated sodium hyaluronate (MHA) and thiolated sodium hyaluronate (SHHA) [217]. Figure S3. The chemical structure of methacrylated gelatin (GelMA) [218]. Figure S4. Structure of methacrylated chitosan [219]. Figure S5. Structure of poly(ethylene glycol) diacrylate (PEGDA). Figure S6. Structure of dimethyl acrylamide (DMAAm). Figure S7. Structure of methylene bis-acrylamide (MBAAm). Figure S8. Structure of ninylpyrrolidone (NVP). Figure S9. Structure of gelatin [220]. Figure S10. Synthetic routine of allyl glycidyl ether (AGE) modified gelatin [119]. Figure S11. Structure of chitosan. Figure S12. Structure of glycol chitosan (GC) [84]. Figure S13. Structure of methacrylated GC (MeGC) [128]. Figure S14. Structure of alginate [221]. Figure S15. Synthetic routine of methacrylated alginate (Alg-MA) treating the secondary hydroxyl groups with MAA [132]. Figure S16. The synthetic routine of oxidized and methacrylated alginates (OMA) [133]. Figure S17. Structure of norbornene functionalized alginate [134]. Figure S18. Structure of phenyl group functionalized alginate (Alg-Ph) [133]. Figure S19. Structure of RGD Peptide Sequence (CGGGRGDS) [135]. Figure S20. Structure of hyaluronan N-acetyl-D-glucosamine and β-D-glucuronic acid linked by β-1,3 and β-1,4 glycosidic bonds. Figure s21. Structure of dopamine-conjugated maleic hyaluronic acid (DMHA) [149]. Figure S22. Structure of tyramine-functionalized hyaluronic acid (HA-Tyr) [222].

## References

[1] Langer R. (2000). Biomaterials in drug delivery and tissue engineering: One laboratory’s experience. Acc. Chem. Res..

[2] Sahara M. (2023). Recent Advances in Generation of In Vitro Cardiac Organoids. Int. J. Mol. Sci..

[3] Gaharwar A.K., Singh I., Khademhosseini A. (2020). Engineered biomaterials for in situ tissue regeneration. Nat. Rev. Mater..

[4] Bayes-Genis A., Galvez-Monton C., Roura S. (2016). Cardiac Tissue Engineering: Lost in Translation or Ready for Translation?. J. Am. Coll. Cardiol..

[5] Acri T.M., Shin K., Seol D., Laird N.Z., Song I., Geary S.M., Chakka J.L., Martin J.A., Salem A.K. (2019). Tissue Engineering for the Temporomandibular Joint. Adv. Healthc. Mater..

[6] Zhao Y., Song S., Ren X., Zhang J., Lin Q., Zhao Y. (2022). Supramolecular Adhesive Hydrogels for Tissue Engineering Applications. Chem. Rev..

[7] Yu C., Schimelman J., Wang P., Miller K.L., Ma X., You S., Guan J., Sun B., Zhu W., Chen S. (2020). Photopolymerizable Biomaterials and Light-Based 3D Printing Strategies for Biomedical Applications. Chem. Rev..

[8] Zhang X., Zhang Y., Ma G., Yang D., Nie J. (2015). The effect of the prefrozen process on properties of a chitosan/hydroxyapatite/poly(methyl methacrylate) composite prepared by freeze drying method used for bone tissue engineering. RSC Adv..

[9] Luo C.J., Stoyanov S.D., Stride E., Pelan E., Edirisinghe M. (2012). Electrospinning versus fibre production methods: From specifics to technological convergence. Chem. Soc. Rev..

[10] Onder O.C., Batool S.R., Nazeer M.A. (2022). Self-assembled silk fibroin hydrogels: From preparation to biomedical applications. Mater. Adv..

[11] Murphy S.V., Atala A. (2014). 3D bioprinting of tissues and organs. Nat. Biotechnol..

[12] Jang J., Yi H.G., Cho D.W. (2016). 3D Printed Tissue Models: Present and Future. ACS Biomater. Sci. Eng..

[13] Ali N.B., Khlif M., Hammami D., Bradai C. (2021). Optimization of structural parameters on hollow spherical cells manufactured by Fused Deposition Modeling (FDM) using Taguchi method. Cell. Polym..

[14] Wang Z., Song Q., Wu H., Feng B., Li Y., Bu L. (2023). Synchronized 3D Printing and Corona Charging for One-Step Prototyping of Polarized Polylactic Acid Electrets. Polymers.

[15] Kačarević Ž., Rider P., Alkildani S., Retnasingh S., Smeets R., Jung O., Ivanišević Z., Barbeck M. (2018). An Introduction to 3D Bioprinting: Possibilities, Challenges and Future Aspects. Materials.

[16] Najihi I., Ennawaoui C., Hajjaji A., Boughaleb Y. (2023). Exploring the piezoelectric porous polymers for energy harvesting: A review. Energy Harvest. Syst..

[17] Palaniyappan S., Veeman D., Sivakumar N.K., Natrayan L. (2022). Development and optimization of lattice structure on the walnut shell reinforced PLA composite for the tensile strength and dimensional error properties. Structures.

[18] Jorgensen A.M., Yoo J.J., Atala A. (2020). Solid Organ Bioprinting: Strategies to Achieve Organ Function. Chem. Rev..

[19] Shin S.R., Zihlmann C., Akbari M., Assawes P., Cheung L., Zhang K., Manoharan V., Zhang Y.S., Yuksekkaya M., Wan K.T. (2016). Reduced Graphene Oxide-GelMA Hybrid Hydrogels as Scaffolds for Cardiac Tissue Engineering. Small.

[20] Lim S.H., Kathuria H., Tan J.J.Y., Kang L. (2018). 3D printed drug delivery and testing systems—A passing fad or the future?. Adv. Drug Deliv. Rev..

[21] Sun Z. (2020). Clinical Applications of Patient-Specific 3D Printed Models in Cardiovascular Disease: Current Status and Future Directions. Biomolecules.

[22] Oh K.C., Park J.M., Shim J.S., Kim J.H., Kim J.E., Kim J.H. (2019). Assessment of metal sleeve-free 3D-printed implant surgical guides. Dent. Mater..

[23] Najihi I., Ennawaoui C., Hajjaji A., Boughaleb Y. (2022). 3D printed cellular piezoelectric polymers for smart sensors/autonomous energy harvesters. Mater. Today Proc..

[24] Mueller E., Poulin I., Bodnaryk W.J., Hoare T. (2022). Click Chemistry Hydrogels for Extrusion Bioprinting: Progress, Challenges, and Opportunities. Biomacromolecules.

[25] Miri A.K., Mirzaee I., Hassan S., Mesbah Oskui S., Nieto D., Khademhosseini A., Zhang Y.S. (2019). Effective bioprinting resolution in tissue model fabrication. Lab. Chip.

[26] Li X., Liu B., Pei B., Chen J., Zhou D., Peng J., Zhang X., Jia W., Xu T. (2020). Inkjet Bioprinting of Biomaterials. Chem. Rev..

[27] Piironen K., Haapala M., Talman V., Jarvinen P., Sikanen T. (2020). Cell adhesion and proliferation on common 3D printing materials used in stereolithography of microfluidic devices. Lab. Chip.

[28] Fang Z., Shi Y., Zhang Y., Zhao Q., Wu J. (2021). Reconfigurable Polymer Networks for Digital Light Processing 3D Printing. ACS Appl. Mater. Interfaces.

[29] Huang Z., Chi-Pong Tsui G., Deng Y., Tang C.-Y. (2020). Two-photon polymerization nanolithography technology for fabrication of stimulus-responsive micro/nano-structures for biomedical applications. Nanotechnol. Rev..

[30] GhavamiNejad A., Ashammakhi N., Wu X.Y., Khademhosseini A. (2020). Crosslinking Strategies for 3D Bioprinting of Polymeric Hydrogels. Small.

[31] Zennifer A., Manivannan S., Sethuraman S., Kumbar S.G., Sundaramurthi D. (2022). 3D bioprinting and photocrosslinking: Emerging strategies & future perspectives. Biomater. Adv..

[32] Lim K.S., Galarraga J.H., Cui X., Lindberg G.C.J., Burdick J.A., Woodfield T.B.F. (2020). Fundamentals and Applications of Photo-Cross-Linking in Bioprinting. Chem. Rev..

[33] Bagheri A., Jin J. (2019). Photopolymerization in 3D Printing. ACS Appl. Polym. Mater..

[34] Lim K.S., Schon B.S., Mekhileri N.V., Brown G.C.J., Chia C.M., Prabakar S., Hooper G.J., Woodfield T.B.F. (2016). New Visible-Light Photoinitiating System for Improved Print Fidelity in Gelatin-Based Bioinks. ACS Biomater. Sci. Eng..

[35] Yagci Y., Jockusch S., Turro N.J. (2010). Photoinitiated Polymerization: Advances, Challenges, and Opportunities. Macromolecules.

[36] Chartrain N.A., Williams C.B., Whittington A.R. (2018). A review on fabricating tissue scaffolds using vat photopolymerization. Acta Biomater..

[37] Zhang F., Zhu L., Li Z., Wang S., Shi J., Tang W., Li N., Yang J. (2021). The recent development of vat photopolymerization: A review. Addit. Manuf..

[38] Yu C., Ma X., Zhu W., Wang P., Miller K.L., Stupin J., Koroleva-Maharajh A., Hairabedian A., Chen S. (2019). Scanningless and continuous 3D bioprinting of human tissues with decellularized extracellular matrix. Biomaterials.

[39] Bejleri D., Streeter B.W., Nachlas A.L.Y., Brown M.E., Gaetani R., Christman K.L., Davis M.E. (2018). A Bioprinted Cardiac Patch Composed of Cardiac-Specific Extracellular Matrix and Progenitor Cells for Heart Repair. Adv. Healthc. Mater..

[40] Grigoryan B., Paulsen S.J., Corbett D.C., Sazer D.W., Fortin C.L., Zaita A.J., Greenfield P.T., Calafat N.J., Gounley J.P., Ta A.H. (2019). Multivascular networks and functional intravascular topologies within biocompatible hydrogels. Science.

[41] Zheng F., Derby B., Wong J. (2021). Fabrication of microvascular constructs using high resolution electrohydrodynamic inkjet printing. Biofabrication.

[42] Zheng Z., Eglin D., Alini M., Richards G.R., Qin L., Lai Y. (2021). Visible Light-Induced 3D Bioprinting Technologies and Corresponding Bioink Materials for Tissue Engineering: A Review. Engineering.

[43] Zaupa A., Terraza C., Abarzua-Illanes P.N., Byres N., Zavala G., Cuenca J., Hidalgo C., Viafara-Garcia S.M., Wolf B., Pino-Lagos K. (2023). A Psychrophilic GelMA: Breaking Technical and Immunological Barriers for Multimaterial High-Resolution 3D Bioprinting. Biomacromolecules.

[44] Scoutaris N., Ross S., Douroumis D. (2016). Current Trends on Medical and Pharmaceutical Applications of Inkjet Printing Technology. Pharm. Res..

[45] Roth E.A., Xu T., Das M., Gregory C., Hickman J.J., Boland T. (2004). Inkjet printing for high-throughput cell patterning. Biomaterials.

[46] Mugnaini G., Resta C., Poggi G., Bonini M. (2021). Photopolymerizable pullulan: Synthesis, self-assembly and inkjet printing. J. Colloid. Interface Sci..

[47] Tigner T.J., Rajput S., Gaharwar A.K., Alge D.L. (2020). Comparison of Photo Cross Linkable Gelatin Derivatives and Initiators for Three-Dimensional Extrusion Bioprinting. Biomacromolecules.

[48] Compaan A.M., Song K., Huang Y. (2019). Gellan Fluid Gel as a Versatile Support Bath Material for Fluid Extrusion Bioprinting. ACS Appl. Mater. Interfaces.

[49] Derakhshanfar S., Mbeleck R., Xu K., Zhang X., Zhong W., Xing M. (2018). 3D bioprinting for biomedical devices and tissue engineering: A review of recent trends and advances. Bioact. Mater..

[50] Kyle S., Jessop Z.M., Al-Sabah A., Whitaker I.S. (2017). ‘Printability’ of Candidate Biomaterials for Extrusion Based 3D Printing: State-of-the-Art. Adv. Healthc. Mater..

[51] Malda J., Visser J., Melchels F.P., Jungst T., Hennink W.E., Dhert W.J., Groll J., Hutmacher D.W. (2013). 25th anniversary article: Engineering hydrogels for biofabrication. Adv. Mater..

[52] Levato R., Jungst T., Scheuring R.G., Blunk T., Groll J., Malda J. (2020). From Shape to Function: The Next Step in Bioprinting. Adv. Mater..

[53] Zhang J., Hu Q., Wang S., Tao J., Gou M. (2020). Digital Light Processing Based Three-dimensional Printing for Medical Applications. Int. J. Bioprint.

[54] Ouyang L., Highley C.B., Sun W., Burdick J.A. (2016). A Generalizable Strategy for the 3D Bioprinting of Hydrogels from Nonviscous Photo-crosslinkable Inks. Adv. Mater..

[55] Lee A., Hudson A.R., Shiwarski D.J., Tashman J.W., Hinton T.J., Yerneni S., Bliley J.M., Campbell P.G., Feinberg A.W. (2019). 3D bioprinting of collagen to rebuild components of the human heart. Science.

[56] Bhattacharjee T., Zehnder S.M., Rowe K.G., Jain S., Nixon R.M., Sawyer W.G., Angelini T.E. (2015). Writing in the granular gel medium. Sci. Adv..

[57] Wu W., DeConinck A., Lewis J.A. (2011). Omnidirectional printing of 3D microvascular networks. Adv. Mater..

[58] Dhariwala B., Hunt E., Boland T. (2004). Rapid prototyping of tissue-engineering constructs, using photopolymerizable hydrogels and stereolithography. Tissue Eng..

[59] Wang Z., Kumar H., Tian Z., Jin X., Holzman J.F., Menard F., Kim K. (2018). Visible Light Photoinitiation of Cell-Adhesive Gelatin Methacryloyl Hydrogels for Stereolithography 3D Bioprinting. ACS Appl. Mater. Interfaces.

[60] Lam T., Dehne T., Kruger J.P., Hondke S., Endres M., Thomas A., Lauster R., Sittinger M., Kloke L. (2019). Photopolymerizable gelatin and hyaluronic acid for stereolithographic 3D bioprinting of tissue-engineered cartilage. J. Biomed. Mater. Res. B Appl. Biomater..

[61] Melhem M.R., Park J., Knapp L., Reinkensmeyer L., Cvetkovic C., Flewellyn J., Lee M.K., Jensen T.W., Bashir R., Kong H. (2017). 3D Printed Stem-Cell-Laden, Microchanneled Hydrogel Patch for the Enhanced Release of Cell-Secreting Factors and Treatment of Myocardial Infarctions. ACS Biomater. Sci. Eng..

[62] Li W., Mille L.S., Robledo J.A., Uribe T., Huerta V., Zhang Y.S. (2020). Recent Advances in Formulating and Processing Biomaterial Inks for Vat Polymerization-Based 3D Printing. Adv. Healthc. Mater..

[63] Goodarzi Hosseinabadi H., Dogan E., Miri A.K., Ionov L. (2022). Digital Light Processing Bioprinting Advances for Microtissue Models. ACS Biomater. Sci. Eng..

[64] Li W., Wang M., Ma H., Chapa-Villarreal F.A., Lobo A.O., Zhang Y.S. (2023). Stereolithography apparatus and digital light processing-based 3D bioprinting for tissue fabrication. iScience.

[65] Gong J., Qian Y., Lu K., Zhu Z., Siow L., Zhang C., Zhou S., Gu T., Yin J., Yu M. (2022). Digital light processing (DLP) in tissue engineering: From promise to reality, and perspectives. Biomed. Mater..

[66] Ma X., Qu X., Zhu W., Li Y.S., Yuan S., Zhang H., Liu J., Wang P., Lai C.S., Zanella F. (2016). Deterministically patterned biomimetic human iPSC-derived hepatic model via rapid 3D bioprinting. Proc. Natl. Acad. Sci. USA.

[67] Ma X., Yu C., Wang P., Xu W., Wan X., Lai C.S.E., Liu J., Koroleva-Maharajh A., Chen S. (2018). Rapid 3D bioprinting of decellularized extracellular matrix with regionally varied mechanical properties and biomimetic microarchitecture. Biomaterials.

[68] Kelly B.E., Bhattacharya I., Heidari H., Shusteff M., Spadaccini C.M., Taylor H.K. (2019). Volumetric additive manufacturing via tomographic reconstruction. Science.

[69] Gungor-Ozkerim P.S., Inci I., Zhang Y.S., Khademhosseini A., Dokmeci M.R. (2018). Bioinks for 3D bioprinting: An overview. Biomater. Sci..

[70] Hull S.M., Lou J., Lindsay C.D., Navarro R.S., Cai B., Brunel L.G., Westerfield A.D., Xia Y., Heilshorn S.C. (2023). 3D bioprinting of dynamic hydrogel bioinks enabled by small molecule modulators. Sci. Adv..

[71] Groll J., Burdick J.A., Cho D.W., Derby B., Gelinsky M., Heilshorn S.C., Jungst T., Malda J., Mironov V.A., Nakayama K. (2018). A definition of bioinks and their distinction from biomaterial inks. Biofabrication.

[72] Li T., Chang J., Zhu Y., Wu C. (2020). 3D Printing of Bioinspired Biomaterials for Tissue Regeneration. Adv. Healthc. Mater..

[73] Li Y., Zhang X., Yan Z., Du L., Tang W., Phillips D.L. (2020). Photochemical alpha-Cleavage Reaction of 3′,5′-Dimethoxybenzoin: A Combined Time-Resolved Spectroscopy and Computational Chemistry Study. Molecules.

[74] Yao M., Liu S., Huang C., Nie J., He Y. (2021). Significantly improve the photoinitiation ability of hydroxyalkyl-derived polymerizable α-hydroxyalkylacetophenone photoinitiators by blocking hyperconjugation. J. Photochem. Photobiol. A Chem..

[75] Gonsalvi L., Peruzzini M. (2012). Novel synthetic pathways for bis(acyl)phosphine oxide photoinitiators. Angew. Chem. Int. Ed. Engl..

[76] Dietlin C., Trinh T.T., Schweizer S., Graff B., Morlet-Savary F., Noirot P.A., Lalevee J. (2020). New Phosphine Oxides as High Performance Near—UV Type I Photoinitiators of Radical Polymerization. Molecules.

[77] Nehlig E., Schneider R., Vidal L., Clavier G., Balan L. (2012). Silver nanoparticles coated with thioxanthone derivative as hybrid photoinitiating systems for free radical polymerization. Langmuir.

[78] Hola E., Fiedor P., Dzienia A., Ortyl J. (2021). Visible-Light Amine Thioxanthone Derivatives as Photoredox Catalysts for Photopolymerization Processes. ACS Appl. Polym. Mater..

[79] Pérez-Mondragón A.A., Cuevas-Suárez C.E., González-López J.A., Trejo-Carbajal N., Herrera-González A.M. (2020). Evaluation of new coinitiators of camphorquinone useful in the radical photopolymerization of dental monomers. J. Photochem. Photobiol. A Chem..

[80] Lin Y.-C., Nixon E.J., Wang H.-Y., Dhawan U., Huang Y.-W., Huang S.-H., Jiang C.-P., Kuo Y.-J., Chung R.-J. (2023). Photocrosslinked Gelatin Methacryloyl (GelMA)/Hyaluronic Acid Methacryloyl (HAMA) Composite Scaffold Using Anthocyanidin as a Photoinitiator for Bone Tissue Regeneration. ACS Appl. Polym. Mater..

[81] Montazerian H., Baidya A., Haghniaz R., Davoodi E., Ahadian S., Annabi N., Khademhosseini A., Weiss P.S. (2021). Stretchable and Bioadhesive Gelatin Methacryloyl-Based Hydrogels Enabled by in Situ Dopamine Polymerization. ACS Appl. Mater. Interfaces.

[82] De Moor L., Smet J., Plovyt M., Bekaert B., Vercruysse C., Asadian M., De Geyter N., Van Vlierberghe S., Dubruel P., Declercq H. (2021). Engineering microvasculature by 3D bioprinting of prevascularized spheroids in photo-crosslinkable gelatin. Biofabrication.

[83] Zhong C., Wu J., Reinhart-King C.A., Chu C.C. (2010). Synthesis, characterization and cytotoxicity of photo-crosslinked maleic chitosan-polyethylene glycol diacrylate hybrid hydrogels. Acta Biomater..

[84] Amsden B.G., Sukarto A., Knight D.K., Shapka S.N. (2007). Methacrylated glycol chitosan as a photopolymerizable biomaterial. Biomacromolecules.

[85] Han W.T., Jang T., Chen S., Chong L.S.H., Jung H.D., Song J. (2019). Improved cell viability for large-scale biofabrication with photo-crosslinkable hydrogel systems through a dual-photoinitiator approach. Biomater. Sci..

[86] Benedikt S., Wang J., Markovic M., Moszner N., Dietliker K., Ovsianikov A., Grützmacher H., Liska R. (2016). Highly efficient water-soluble visible light photoinitiators. J. Polym. Sci. Part A Polym. Chem..

[87] Kunwar P., Jannini A.V.S., Xiong Z., Ransbottom M.J., Perkins J.S., Henderson J.H., Hasenwinkel J.M., Soman P. (2020). High-Resolution 3D Printing of Stretchable Hydrogel Structures Using Optical Projection Lithography. ACS Appl. Mater. Interfaces.

[88] Ghazali H.S., Askari E., Seyfoori A., Naghib S.M. (2023). A high-absorbance water-soluble photoinitiator nanoparticle for hydrogel 3D printing: Synthesis, characterization and in vitro cytotoxicity study. Sci. Rep..

[89] Mirdamadi E., Tashman J.W., Shiwarski D.J., Palchesko R.N., Feinberg A.W. (2020). FRESH 3D Bioprinting a Full-Size Model of the Human Heart. ACS Biomater. Sci. Eng..

[90] Mirdamadi E., Muselimyan N., Koti P., Asfour H., Sarvazyan N. (2019). Agarose Slurry as a Support Medium for Bioprinting and Culturing Freestanding Cell-Laden Hydrogel Constructs. 3D Print. Addit. Manuf..

[91] Bernal P.N., Delrot P., Loterie D., Li Y., Malda J., Moser C., Levato R. (2019). Volumetric Bioprinting of Complex Living-Tissue Constructs within Seconds. Adv. Mater..

[92] Jakubiak J., Allonas X., Fouassier J.P., Sionkowska A., Andrzejewska E., Linden L.Å., Rabek J.F. (2003). Camphorquinone–amines photoinitating systems for the initiation of free radical polymerization. Polymer.

[93] Kirschner J., Szillat F., Bouzrati-Zerelli M., Becht J.M., Klee J.E., Lalevee J. (2020). Sulfinates and sulfonates as high performance co-initiators in CQ based systems: Towards aromatic amine-free systems for dental restorative materials. Dent. Mater..

[94] Kowsari K., Lee W., Yoo S.-S., Fang N.X. (2021). Scalable visible light 3D printing and bioprinting using an organic light-emitting diode microdisplay. iScience.

[95] Tomal W., Ortyl J. (2020). Water-Soluble Photoinitiators in Biomedical Applications. Polymers.

[96] Kamoun E.A., El-Betany A., Menzel H., Chen X. (2018). Influence of photoinitiator concentration and irradiation time on the crosslinking performance of visible-light activated pullulan-HEMA hydrogels. Int. J. Biol. Macromol..

[97] Chen L., Kenkel S.M., Hsieh P.H., Gryka M.C., Bhargava R. (2020). Freeform Three-Dimensionally Printed Microchannels via Surface-Initiated Photopolymerization Combined with Sacrificial Molding. ACS Appl. Mater. Interfaces.

[98] Petta D., Grijpma D.W., Alini M., Eglin D., D’Este M. (2018). Three-Dimensional Printing of a Tyramine Hyaluronan Derivative with Double Gelation Mechanism for Independent Tuning of Shear Thinning and Postprinting Curing. ACS Biomater. Sci. Eng..

[99] Piluso S., Flores Gomez D., Dokter I., Moreira Texeira L., Li Y., Leijten J., van Weeren R., Vermonden T., Karperien M., Malda J. (2020). Rapid and cytocompatible cell-laden silk hydrogel formation via riboflavin-mediated crosslinking. J. Mater. Chem. B.

[100] Shrestha A., Hamblin M.R., Kishen A. (2014). Photoactivated rose bengal functionalized chitosan nanoparticles produce antibacterial/biofilm activity and stabilize dentin-collagen. Nanomedicine.

[101] Caprioli M., Roppolo I., Chiappone A., Larush L., Pirri C.F., Magdassi S. (2021). 3D-printed self-healing hydrogels via Digital Light Processing. Nat. Commun..

[102] Nur Karakus F., Bulgurcuoglu Kuran S., Solakoglu S. (2021). Effect of curcumin on sperm parameters after the cryopreservation. Eur. J. Obstet. Gynecol. Reprod. Biol..

[103] Lin F., Fan W., Wise G.E. (1991). Eosin Y staining of proteins in polyacrylamide gels. Anal. Biochem..

[104] Fouassier J.P., Chesneau E. (1991). Polymérisation induite sous irradiation laser visible, 4†. Le système éosine/photoamorceur ultra-violet/amine. Die Makromol. Chem..

[105] Aguirre-Soto A., Kim S., Kaastrup K., Sikes H.D. (2019). On the role of N-vinylpyrrolidone in the aqueous radical-initiated copolymerization with PEGDA mediated by eosin Y in the presence of O_2_. Polym. Chem..

[106] Hao Y., Shih H., Muňoz Z., Kemp A., Lin C.-C. (2014). Visible light cured thiol-vinyl hydrogels with tunable degradation for 3D cell culture. Acta Biomater..

[107] Kim E., Kim M.H., Song J.H., Kang C., Park W.H. (2020). Dual crosslinked alginate hydrogels by riboflavin as photoinitiator. Int. J. Biol. Macromol..

[108] Bertolotti S.G., Previtali C.M., Rufs A.M., Encinas M.V. (1999). Riboflavin/Triethanolamine as Photoinitiator System of Vinyl Polymerization. A Mechanistic Study by Laser Flash Photolysis. Macromolecules.

[109] Grotzinger C., Burget D., Jacques P., Fouassier J.P. (2003). Visible light induced photopolymerization: Speeding up the rate of polymerization by using co-initiators in dye/amine photoinitiating systems. Polymer.

[110] Fischer B.B., Krieger-Liszkay A., Eggen R.L. (2004). Photosensitizers neutral red (type I) and rose bengal (type II) cause light-dependent toxicity in Chlamydomonas reinhardtii and induce the Gpxh gene via increased singlet oxygen formation. Environ. Sci. Technol..

[111] Ahn D., Stevens L.M., Zhou K., Page Z.A. (2020). Rapid High-Resolution Visible Light 3D Printing. ACS Cent. Sci..

[112] Balzani V., Ceroni P., Credi A., Venturi M. (2021). Ruthenium tris(bipyridine) complexes: Interchange between photons and electrons in molecular-scale devices and machines. Coord. Chem. Rev..

[113] Fancy D.A., Kodadek T. (1999). Chemistry for the analysis of protein–protein interactions: Rapid and efficient cross-linking triggered by long wavelength light. Proc. Natl. Acad. Sci. USA.

[114] Shaukat U., Rossegger E., Schlogl S. (2022). A Review of Multi-Material 3D Printing of Functional Materials via Vat Photopolymerization. Polymers.

[115] Van Den Bulcke A.I., Bogdanov B., De Rooze N., Schacht E.H., Cornelissen M., Berghmans H. (2000). Structural and rheological properties of methacrylamide modified gelatin hydrogels. Biomacromolecules.

[116] Leu Alexa R., Cucuruz A., Ghitulica C.D., Voicu G., Stamat Balahura L.R., Dinescu S., Vlasceanu G.M., Stavarache C., Ianchis R., Iovu H. (2022). 3D Printable Composite Biomaterials Based on GelMA and Hydroxyapatite Powders Doped with Cerium Ions for Bone Tissue Regeneration. Int. J. Mol. Sci..

[117] Man K., Barroso I.A., Brunet M.Y., Peacock B., Federici A.S., Hoey D.A., Cox S.C. (2022). Controlled Release of Epigenetically-Enhanced Extracellular Vesicles from a GelMA/Nanoclay Composite Hydrogel to Promote Bone Repair. Int. J. Mol. Sci..

[118] Bertassoni L.E., Cardoso J.C., Manoharan V., Cristino A.L., Bhise N.S., Araujo W.A., Zorlutuna P., Vrana N.E., Ghaemmaghami A.M., Dokmeci M.R. (2014). Direct-write bioprinting of cell-laden methacrylated gelatin hydrogels. Biofabrication.

[119] Bertlein S., Brown G., Lim K.S., Jungst T., Boeck T., Blunk T., Tessmar J., Hooper G.J., Woodfield T.B.F., Groll J. (2017). Thiol-Ene Clickable Gelatin: A Platform Bioink for Multiple 3D Biofabrication Technologies. Adv. Mater..

[120] Peers S., Montembault A., Ladaviere C. (2020). Chitosan hydrogels for sustained drug delivery. J. Control. Release.

[121] Peers S., Montembault A., Ladavière C. (2022). Chitosan hydrogels incorporating colloids for sustained drug delivery. Carbohydr. Polym..

[122] Deng L., Wang L., Li L., Gong Z., Wang R., Fei W., Zhou Y., Wang F. (2023). Bioabsorbable Fibrillar Gauze Dressing Based on N-Carboxyethyl Chitosan Gelling Fibers for Fatal Hemorrhage Control. ACS Appl. Bio Mater..

[123] Zhang R., Chang S.J., Jing Y., Wang L., Chen C.J., Liu J.T. (2023). Application of chitosan with different molecular weights in cartilage tissue engineering. Carbohydr. Polym..

[124] Wang X.-Y., Wang J., Rousseau D., Tang C.-H. (2023). Fabrication of chitosan colloidal gels via pH-mediated self-association. Food Hydrocolloids.

[125] Rajabi M., McConnell M., Cabral J., Ali M.A. (2021). Chitosan hydrogels in 3D printing for biomedical applications. Carbohydr. Polym..

[126] Taghizadeh M., Taghizadeh A., Yazdi M.K., Zarrintaj P., Stadler F.J., Ramsey J.D., Habibzadeh S., Hosseini Rad S., Naderi G., Saeb M.R. (2022). Chitosan-based inks for 3D printing and bioprinting. Green. Chem..

[127] Mallakpour S., Sirous F., Hussain C.M. (2021). Current achievements in 3D bioprinting technology of chitosan and its hybrids. New J. Chem..

[128] Yang D.H., Seo D.I., Lee D.-W., Bhang S.H., Park K., Jang G., Kim C.H., Chun H.J. (2017). Preparation and evaluation of visible-light cured glycol chitosan hydrogel dressing containing dual growth factors for accelerated wound healing. J. Ind. Eng. Chem..

[129] Cao Y., Cong H., Yu B., Shen Y. (2023). A review on the synthesis and development of alginate hydrogels for wound therapy. J. Mater. Chem. B.

[130] Mallakpour S., Azadi E., Hussain C.M. (2021). State-of-the-art of 3D printing technology of alginate-based hydrogels-An emerging technique for industrial applications. Adv. Colloid. Interface Sci..

[131] Sun J.Y., Zhao X., Illeperuma W.R., Chaudhuri O., Oh K.H., Mooney D.J., Vlassak J.J., Suo Z. (2012). Highly stretchable and tough hydrogels. Nature.

[132] Hasany M., Talebian S., Sadat S., Ranjbar N., Mehrali M., Wallace G.G., Mehrali M. (2021). Synthesis, properties, and biomedical applications of alginate methacrylate (ALMA)-based hydrogels: Current advances and challenges. Appl. Mater. Today.

[133] Jeon O., Alt D.S., Ahmed S.M., Alsberg E. (2012). The effect of oxidation on the degradation of photocrosslinkable alginate hydrogels. Biomaterials.

[134] Ooi H.W., Mota C., Ten Cate A.T., Calore A., Moroni L., Baker M.B. (2018). Thiol-Ene Alginate Hydrogels as Versatile Bioinks for Bioprinting. Biomacromolecules.

[135] Sakai S., Komatani K., Taya M. (2012). Glucose-triggered co-enzymatic hydrogelation of aqueous polymer solutions. RSC Adv..

[136] Khanmohammadi M., Sakai S., Taya M. (2019). Characterization of encapsulated cells within hyaluronic acid and alginate microcapsules produced via horseradish peroxidase-catalyzed crosslinking. J. Biomater. Sci. Polym. Ed..

[137] Ramanathan R.K., McDonough S.L., Philip P.A., Hingorani S.R., Lacy J., Kortmansky J.S., Thumar J., Chiorean E.G., Shields A.F., Behl D. (2019). Phase IB/II Randomized Study of FOLFIRINOX Plus Pegylated Recombinant Human Hyaluronidase Versus FOLFIRINOX Alone in Patients with Metastatic Pancreatic Adenocarcinoma: SWOG S1313. J. Clin. Oncol..

[138] Kim H., Shin M., Han S., Kwon W., Hahn S.K. (2019). Hyaluronic Acid Derivatives for Translational Medicines. Biomacromolecules.

[139] Gaffney J., Matou-Nasri S., Grau-Olivares M., Slevin M. (2010). Therapeutic applications of hyaluronan. Mol. Biosyst..

[140] Chaudhry G.E., Akim A., Naveed Zafar M., Safdar N., Sung Y.Y., Muhammad T.S.T. (2021). Understanding Hyaluronan Receptor (CD44) Interaction, HA-CD44 Activated Potential Targets in Cancer Therapeutics. Adv. Pharm. Bull..

[141] Kim S.J., Owen S.C. (2020). Hyaluronic acid binding to CD44S is indiscriminate of molecular weight. Biochim. Biophys. Acta Biomembr..

[142] Hofinger E.S., Hoechstetter J., Oettl M., Bernhardt G., Buschauer A. (2008). Isoenzyme-specific differences in the degradation of hyaluronic acid by mammalian-type hyaluronidases. Glycoconj. J..

[143] Shigefuji M., Tokudome Y. (2020). Nanoparticulation of hyaluronic acid: A new skin penetration enhancing polyion complex formulation: Mechanism and future potential. Materialia.

[144] Snetkov P., Zakharova K., Morozkina S., Olekhnovich R., Uspenskaya M. (2020). Hyaluronic Acid: The Influence of Molecular Weight on Structural, Physical, Physico-Chemical, and Degradable Properties of Biopolymer. Polymers.

[145] Chaudhari A.A., Vig K., Baganizi D.R., Sahu R., Dixit S., Dennis V., Singh S.R., Pillai S.R. (2016). Future Prospects for Scaffolding Methods and Biomaterials in Skin Tissue Engineering: A Review. Int. J. Mol. Sci..

[146] Wang Y., Liu G., Wu L., Qu H., Song D., Huang H., Wu C., Xu M. (2020). Rational design of porous starch/hyaluronic acid composites for hemostasis. Int. J. Biol. Macromol..

[147] Smeds K.A., Grinstaff M.W. (2001). Photocrosslinkable polysaccharides forin situ hydrogel formation. J. Biomed. Mater. Res..

[148] Bencherif S.A., Srinivasan A., Horkay F., Hollinger J.O., Matyjaszewski K., Washburn N.R. (2008). Influence of the degree of methacrylation on hyaluronic acid hydrogels properties. Biomaterials.

[149] Fan P., Dong Q., Yang J., Chen Y., Yang H., Gu S., Xu W., Zhou Y. (2023). Flexible dual-functionalized hyaluronic acid hydrogel adhesives formed in situ for rapid hemostasis. Carbohydr. Polym..

[150] Loebel C., Broguiere N., Alini M., Zenobi-Wong M., Eglin D. (2015). Microfabrication of Photo-Cross-Linked Hyaluronan Hydrogels by Single- and Two-Photon Tyramine Oxidation. Biomacromolecules.

[151] Cai R., Nakamoto T., Kawazoe N., Chen G. (2015). Influence of stepwise chondrogenesis-mimicking 3D extracellular matrix on chondrogenic differentiation of mesenchymal stem cells. Biomaterials.

[152] Wang X., Chen Z., Zhou B., Duan X., Weng W., Cheng K., Wang H., Lin J. (2018). Cell-Sheet-Derived ECM Coatings and Their Effects on BMSCs Responses. ACS Appl. Mater. Interfaces.

[153] Elliott R.A., Hoehn J.G. (1973). Use of commercial porcine skin for wound dressings. Plast. Reconstr. Surg..

[154] Badylak S.F., Gilbert T.W. (2008). Immune response to biologic scaffold materials. Semin. Immunol..

[155] Spang M.T., Christman K.L. (2018). Extracellular matrix hydrogel therapies: In vivo applications and development. Acta Biomater..

[156] Keane T.J., Dziki J., Sobieski E., Smoulder A., Castleton A., Turner N., White L.J., Badylak S.F. (2017). Restoring Mucosal Barrier Function and Modifying Macrophage Phenotype with an Extracellular Matrix Hydrogel: Potential Therapy for Ulcerative Colitis. J. Crohns Colitis.

[157] Zhang W., Du A., Liu S., Lv M., Chen S. (2021). Research progress in decellularized extracellular matrix-derived hydrogels. Regen. Ther..

[158] De Paula A.G.P., de Lima J.D., Bastos T.S.B., Czaikovski A.P., Dos Santos Luz R.B., Yuasa B.S., Smanioto C.C.S., Robert A.W., Braga T.T. (2023). Decellularized Extracellular Matrix: The Role of This Complex Biomaterial in Regeneration. ACS Omega.

[159] Dabaghi M., Saraei N., Carpio M.B., Nanduri V., Ungureanu J., Babi M., Chandiramohan A., Noble A., Revill S.D., Zhang B. (2021). A Robust Protocol for Decellularized Human Lung Bioink Generation Amenable to 2D and 3D Lung Cell Culture. Cells.

[160] Hoshiba T. (2021). Decellularized Extracellular Matrix for Cell Biology. Curr. Protoc..

[161] Biehl A., Gracioso Martins A.M., Davis Z.G., Sze D., Collins L., Mora-Navarro C., Fisher M.B., Freytes D.O. (2023). Towards a standardized multi-tissue decellularization protocol for the derivation of extracellular matrix materials. Biomater. Sci..

[162] Zhang X., Chen X., Hong H., Hu R., Liu J., Liu C. (2022). Decellularized extracellular matrix scaffolds: Recent trends and emerging strategies in tissue engineering. Bioact. Mater..

[163] Khoshnood N., Zamanian A. (2020). Decellularized extracellular matrix bioinks and their application in skin tissue engineering. Bioprinting.

[164] Bejleri D., Davis M.E. (2019). Decellularized Extracellular Matrix Materials for Cardiac Repair and Regeneration. Adv. Healthc. Mater..

[165] Kim Y.S., Majid M., Melchiorri A.J., Mikos A.G. (2019). Applications of decellularized extracellular matrix in bone and cartilage tissue engineering. Bioeng. Transl. Med..

[166] Li T., Javed R., Ao Q. (2021). Xenogeneic Decellularized Extracellular Matrix-based Biomaterials for Peripheral Nerve Repair and Regeneration. Curr. Neuropharmacol..

[167] Lewis P.L., Su J., Yan M., Meng F., Glaser S.S., Alpini G.D., Green R.M., Sosa-Pineda B., Shah R.N. (2018). Complex bile duct network formation within liver decellularized extracellular matrix hydrogels. Sci. Rep..

[168] Kim J.W., Nam S.A., Yi J., Kim J.Y., Lee J.Y., Park S.Y., Sen T., Choi Y.M., Lee J.Y., Kim H.L. (2022). Kidney Decellularized Extracellular Matrix Enhanced the Vascularization and Maturation of Human Kidney Organoids. Adv. Sci..

[169] Elomaa L., Keshi E., Sauer I.M., Weinhart M. (2020). Development of GelMA/PCL and dECM/PCL resins for 3D printing of acellular in vitro tissue scaffolds by stereolithography. Mater. Sci. Eng. C Mater. Biol. Appl..

[170] Sun Y., Yu K., Nie J., Sun M., Fu J., Wang H., He Y. (2021). Modeling the printability of photocuring and strength adjustable hydrogel bioink during projection-based 3D bioprinting. Biofabrication.

[171] Vurat M.T., Ergun C., Elçin A.E., Elçin Y.M. (2020). 3D Bioprinting of Tissue Models with Customized Bioinks. Adv. Exp. Med. Biol..

[172] Kannurpatti A.R., Anseth J.W., Bowman C.N. (1998). A study of the evolution of mechanical properties and structural heterogeneity of polymer networks formed by photopolymerizations of multifunctional (meth)acrylates. Polymer.

[173] Xin S., Chimene D., Garza J.E., Gaharwar A.K., Alge D.L. (2019). Clickable PEG hydrogel microspheres as building blocks for 3D bioprinting. Biomater. Sci..

[174] Hoyle C.E., Bowman C.N. (2010). Thiol-ene click chemistry. Angew. Chem. Int. Ed. Engl..

[175] Lee B., Lum N., Seow L., Lim P., Tan L. (2016). Synthesis and Characterization of Types A and B Gelatin Methacryloyl for Bioink Applications. Materials.

[176] Rodríguez-Rivero C., Hilliou L., Martín del Valle E.M., Galán M.A. (2014). Rheological characterization of commercial highly viscous alginate solutions in shear and extensional flows. Rheol. Acta.

[177] Van Hoorick J., Gruber P., Markovic M., Tromayer M., Van Erps J., Thienpont H., Liska R., Ovsianikov A., Dubruel P., Van Vlierberghe S. (2017). Cross-Linkable Gelatins with Superior Mechanical Properties Through Carboxylic Acid Modification: Increasing the Two-Photon Polymerization Potential. Biomacromolecules.

[178] Bhattacharyya A., Janarthanan G., Noh I. (2021). Nano-biomaterials for designing functional bioinks towards complex tissue and organ regeneration in 3D bioprinting. Addit. Manuf..

[179] Bernal P.N., Bouwmeester M., Madrid-Wolff J., Falandt M., Florczak S., Rodriguez N.G., Li Y., Grossbacher G., Samsom R.A., van Wolferen M. (2022). Volumetric Bioprinting of Organoids and Optically Tuned Hydrogels to Build Liver-Like Metabolic Biofactories. Adv. Mater..

[180] Han X., Courseaus J., Khamassi J., Nottrodt N., Engelhardt S., Jacobsen F., Bierwisch C., Meyer W., Walter T., Weisser J. (2018). Optimized vascular network by stereolithography for tissue engineered skin. Int. J. Bioprint.

[181] Wang M., Deng Z., Guo Y., Xu P. (2022). Designing functional hyaluronic acid-based hydrogels for cartilage tissue engineering. Mater. Today Bio.

[182] Burdick J.A., Prestwich G.D. (2011). Hyaluronic acid hydrogels for biomedical applications. Adv. Mater..

[183] Sphabmixay P., Raredon M.S.B., Wang A.J., Lee H., Hammond P.T., Fang N.X., Griffith L.G. (2021). High resolution stereolithography fabrication of perfusable scaffolds to enable long-term meso-scale hepatic culture for disease modeling. Biofabrication.

[184] Lewis P.L., Shah R.N. (2016). 3D Printing for Liver Tissue Engineering: Current Approaches and Future Challenges. Curr. Transplant. Rep..

[185] Li W., Liu Z., Tang F., Jiang H., Zhou Z., Hao X., Zhang J.M. (2023). Application of 3D Bioprinting in Liver Diseases. Micromachines.

[186] Frankowski J., Kurzatkowska M., Sobczak M., Piotrowska U. (2023). Utilization of 3D bioprinting technology in creating human tissue and organoid models for preclinical drug research—State-of-the-art. Int. J. Pharm..

[187] Sun L., Wang Y., Zhang S., Yang H., Mao Y. (2023). 3D bioprinted liver tissue and disease models: Current advances and future perspectives. Biomater. Adv..

[188] Kufelt O., El-Tamer A., Sehring C., Schlie-Wolter S., Chichkov B.N. (2014). Hyaluronic acid based materials for scaffolding via two-photon polymerization. Biomacromolecules.

[189] Murphy S.V., De Coppi P., Atala A. (2020). Opportunities and challenges of translational 3D bioprinting. Nat. Biomed. Eng..

[190] Garot C., Bettega G., Picart C. (2021). Additive Manufacturing of Material Scaffolds for Bone Regeneration: Toward Application in the Clinics. Adv. Funct. Mater..

[191] Rajabi M., Cabral J.D., Saunderson S., Ali M.A. (2023). 3D printing of chitooligosaccharide-polyethylene glycol diacrylate hydrogel inks for bone tissue regeneration. J. Biomed. Mater. Res. A.

[192] Ma Y., Ji Y., Zhong T., Wan W., Yang Q., Li A., Zhang X., Lin M. (2017). Bioprinting-Based PDLSC-ECM Screening for in Vivo Repair of Alveolar Bone Defect Using Cell-Laden, Injectable and Photocrosslinkable Hydrogels. ACS Biomater. Sci. Eng..

[193] Jia C., Luo B., Wang H., Bian Y., Li X., Li S., Wang H. (2017). Precise and Arbitrary Deposition of Biomolecules onto Biomimetic Fibrous Matrices for Spatially Controlled Cell Distribution and Functions. Adv. Mater..

[194] Cidonio G., Alcala-Orozco C.R., Lim K.S., Glinka M., Mutreja I., Kim Y.H., Dawson J.I., Woodfield T.B.F., Oreffo R.O.C. (2019). Osteogenic and angiogenic tissue formation in high fidelity nanocomposite Laponite-gelatin bioinks. Biofabrication.

[195] McCormack A., Highley C.B., Leslie N.R., Melchels F.P.W. (2020). 3D Printing in Suspension Baths: Keeping the Promises of Bioprinting Afloat. Trends Biotechnol..

[196] Izadifar M., Chapman D., Babyn P., Chen X., Kelly M.E. (2018). UV-Assisted 3D Bioprinting of Nanoreinforced Hybrid Cardiac Patch for Myocardial Tissue Engineering. Tissue Eng. Part. C Methods.

[197] Maiullari F., Costantini M., Milan M., Pace V., Chirivi M., Maiullari S., Rainer A., Baci D., Marei H.E., Seliktar D. (2018). A multi-cellular 3D bioprinting approach for vascularized heart tissue engineering based on HUVECs and iPSC-derived cardiomyocytes. Sci. Rep..

[198] Costantini M., Testa S., Mozetic P., Barbetta A., Fuoco C., Fornetti E., Tamiro F., Bernardini S., Jaroszewicz J., Swieszkowski W. (2017). Microfluidic-enhanced 3D bioprinting of aligned myoblast-laden hydrogels leads to functionally organized myofibers in vitro and in vivo. Biomaterials.

[199] Barros N.R., Kim H.J., Gouidie M.J., Lee K., Bandaru P., Banton E.A., Sarikhani E., Sun W., Zhang S., Cho H.J. (2021). Biofabrication of endothelial cell, dermal fibroblast, and multilayered keratinocyte layers for skin tissue engineering. Biofabrication.

[200] Zhou F., Hong Y., Liang R., Zhang X., Liao Y., Jiang D., Zhang J., Sheng Z., Xie C., Peng Z. (2020). Rapid printing of bio-inspired 3D tissue constructs for skin regeneration. Biomaterials.

[201] Koch L., Deiwick A., Schlie S., Michael S., Gruene M., Coger V., Zychlinski D., Schambach A., Reimers K., Vogt P.M. (2012). Skin tissue generation by laser cell printing. Biotechnol. Bioeng..

[202] Cui X., Breitenkamp K., Finn M.G., Lotz M., D’Lima D.D. (2012). Direct human cartilage repair using three-dimensional bioprinting technology. Tissue Eng. Part. A.

[203] Phull A.R., Eo S.H., Abbas Q., Ahmed M., Kim S.J. (2016). Applications of Chondrocyte-Based Cartilage Engineering: An Overview. Biomed. Res. Int..

[204] Nakayama N., Pothiawala A., Lee J.Y., Matthias N., Umeda K., Ang B.K., Huard J., Huang Y., Sun D. (2020). Human pluripotent stem cell-derived chondroprogenitors for cartilage tissue engineering. Cell Mol. Life Sci..

[205] Sun A.X., Lin H., Beck A.M., Kilroy E.J., Tuan R.S. (2015). Projection Stereolithographic Fabrication of Human Adipose Stem Cell-Incorporated Biodegradable Scaffolds for Cartilage Tissue Engineering. Front. Bioeng. Biotechnol..

[206] Gao G., Schilling A.F., Hubbell K., Yonezawa T., Truong D., Hong Y., Dai G., Cui X. (2015). Improved properties of bone and cartilage tissue from 3D inkjet-bioprinted human mesenchymal stem cells by simultaneous deposition and photocrosslinking in PEG-GelMA. Biotechnol. Lett..

[207] Mao Q., Wang Y., Li Y., Juengpanich S., Li W., Chen M., Yin J., Fu J., Cai X. (2020). Fabrication of liver microtissue with liver decellularized extracellular matrix (dECM) bioink by digital light processing (DLP) bioprinting. Mater. Sci. Eng. C Mater. Biol. Appl..

[208] Jang J., Kim T.G., Kim B.S., Kim S.W., Kwon S.M., Cho D.W. (2016). Tailoring mechanical properties of decellularized extracellular matrix bioink by vitamin B2-induced photo-crosslinking. Acta Biomater..

[209] Ma Z.Y., Li J., Dong X.H., Cui Y.T., Cui Y.F., Ban T., Huo R. (2023). The role of BRG1 in epigenetic regulation of cardiovascular diseases. Eur. J. Pharmacol..

[210] Ullah M., Bibi A., Wahab A., Hamayun S., Rehman M.U., Khan S.U., Awan U.A., Riaz N.U., Naeem M., Saeed S. (2023). Shaping the Future of Cardiovascular Disease by 3D Printing Applications in Stent Technology and its Clinical Outcomes. Curr. Probl. Cardiol..

[211] Mathes S.H., Ruffner H., Graf-Hausner U. (2014). The use of skin models in drug development. Adv. Drug Deliv. Rev..

[212] Koons G.L., Diba M., Mikos A.G. (2020). Materials design for bone-tissue engineering. Nat. Rev. Mater..

[213] Salhotra A., Shah H.N., Levi B., Longaker M.T. (2020). Mechanisms of bone development and repair. Nat. Rev. Mol. Cell Biol..

[214] Gao C., Peng S., Feng P., Shuai C. (2017). Bone biomaterials and interactions with stem cells. Bone Res..

[215] Rajput M., Mondal P., Yadav P., Chatterjee K. (2022). Light-based 3D bioprinting of bone tissue scaffolds with tunable mechanical properties and architecture from photocurable silk fibroin. Int. J. Biol. Macromol..

[216] Chang H.K., Yang D.H., Ha M.Y., Kim H.J., Kim C.H., Kim S.H., Choi J.W., Chun H.J. (2022). 3D printing of cell-laden visible light curable glycol chitosan bioink for bone tissue engineering. Carbohydr. Polym..

[217] Wan T., Fan P., Zhang M., Shi K., Chen X., Yang H., Liu X., Xu W., Zhou Y. (2022). Multiple Crosslinking Hyaluronic Acid Hydrogels with Improved Strength and 3D Printability. ACS Appl. Bio Mater..

[218] Vigata M., Meinert C., Pahoff S., Bock N., Hutmacher D.W. (2020). Gelatin Methacryloyl Hydrogels Control the Localized Delivery of Albumin-Bound Paclitaxel. Polymers.

[219] Zanon M., Chiappone A., Garino N., Canta M., Frascella F., Hakkarainen M., Pirri C.F., Sangermano M. (2022). Microwave-assisted methacrylation of chitosan for 3D printable hydrogels in tissue engineering. Mater. Adv..

[220] Picard J., Giraudier S., Larreta-Garde V. (2009). Controlled remodeling of a protein-polysaccharide mixed gel: Examples of gelatin-hyaluronic acid mixtures. Soft Matter..

[221] Salisu A., Sanagi M.M., Abu Naim A., Wan Ibrahim W.A., Abd Karim K.J. (2015). Removal of lead ions from aqueous solutions using sodium alginate-graft-poly(methyl methacrylate) beads. Desalination Water Treat..

[222] Loebel C., D’Este M., Alini M., Zenobi-Wong M., Eglin D. (2015). Precise tailoring of tyramine-based hyaluronan hydrogel properties using DMTMM conjugation. Carbohydr. Polym..

