# Supplementary material for: Applications of Light-Based 3D Bioprinting and Photoactive Biomaterials for Tissue Engineering
Source: Materials (Basel). 2023 Nov 30;16(23):7461. doi: 10.3390/ma16237461 (PMC10707029; doi:10.3390/ma16237461)
Supplement: Supplementary file 1 [file materials-16-07461-s001.zip › materials-2593394-supplementary.pdf]

Electronic Supplementary Information

Applications of Light-Based 3D Bioprinting and Photoactive Biomaterials for Tissue Engineering

Xueqin Zhang [1,*], Xin Zhang [1], Ying Li [1] and Yuxuan Zhang [2,*]

[1] College of Chemistry and Materials Engineering, Beijing Technology and Business University, Beijing 100048, China
[2] FuYang Sineva Materials Technology Co., LTD., Beijing 100176, China
* Correspondence: zhangxueqin@btbu.edu.cn (X.Z.); zhangyuxuan@sineva.com.cn (Y.Z.)

CONTENTS

Figure S1. Structure of HAMA S3

Figure S2. Structures of maleiated sodium hyaluronate (MHA) and thiolated sodium hyaluronate (SHHA) [1]..S3

Figure S3. Structure of methacrylated gelatin (GelMA) [2]……..................................S4

Figure S4. Structure of methacrylated chitosan [3]..S4

Figure S5. Structure of poly(ethylene glycol) diacrylate (PEGDA)..........................S4

Figure S6. Structure of dimethyl acrylamide (DMAAm). S5

Figure S7. Structure of methylene bis-acrylamide (MBAAm)................................S5

Figure S8. Structure of ninylpyrrolidone (NVP)………….......................................S5

Figure S9. Structure of gelatin [4]...S6

Figure S10. Synthetic routine of allyl glycidyl ether (AGE) modified gelatin [5]....S6

Figure S11. Structure of chitosan..S6

Figure S12. Structure of glycol chitosan (GC) [6]..S7

Figure S13. Structure of methacrylated GC (MeGC) [7]...S7

Figure S14. Structure of alginate [8]..S7

Figure S15. Synthetic routine of methacrylated alginate (Alg-MA) treating the secondary hydroxyl groups with MAA [9]...S8

Figure S16. The synthetic routine of oxidized and methacrylated alginates (OMA) [10]..S8

Figure S17. Structure of norbornene functionalized alginate [11]..........................S9

Figure S18. Structure of phenyl group functionalized alginate (Alg-Ph) [12]..S9
Figure S19. Structure of RGD Peptide Sequence (CGGGRGDS) [11]..................S9
Figure S20. Structure of hyaluronan N-acetyl-D-glucosamine and β-D-glucuronic acid linked by β-1,3 and β-1,4 glycosidic bonds...S10
Figure S21. Structure of dopamine-conjugated maleic hyaluronic acid (DMHA) [13]..S10
Figure S22. Structure of tyramine-functionalized hyaluronic acid (HA-Tyr) [14].S10
RERERENCES S10

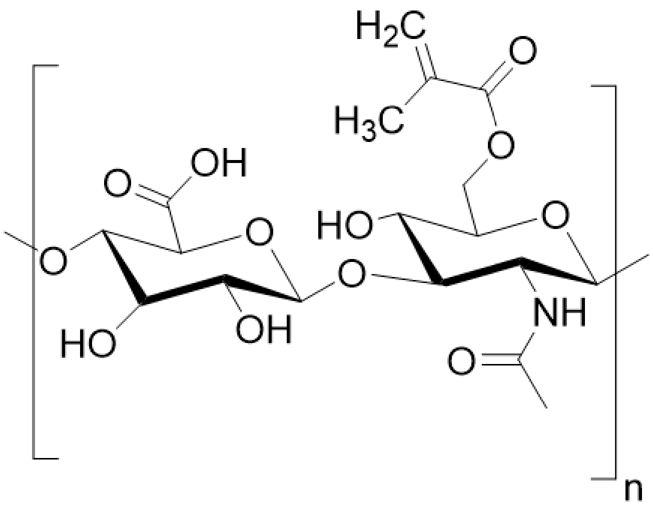

Figure S1. Structure of HAMA.

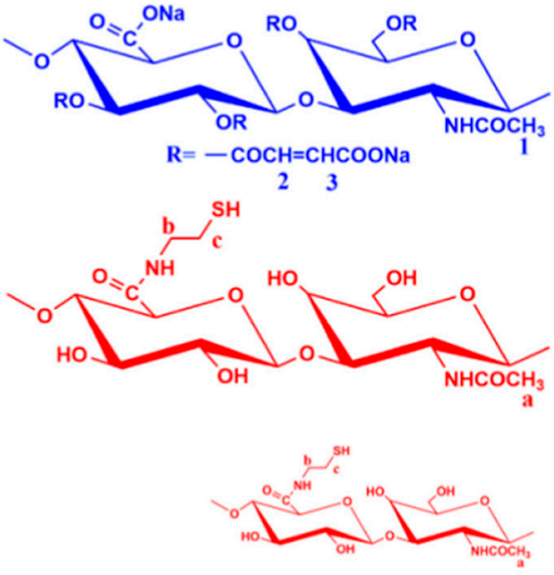

Figure S2. Structures of maleiated sodium hyaluronate (MHA) and thiolated sodium hyaluronate (SHHA) [1].

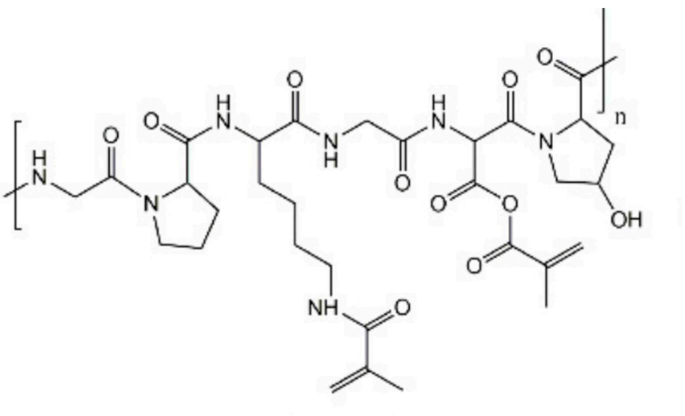

Figure S3. The chemical structure of methacrylated gelatin (GelMA) [2].

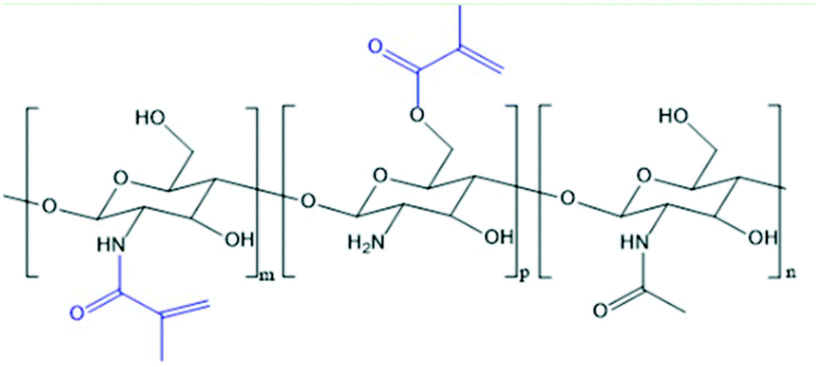

Figure S4. Structure of methacrylated chitosan [3].

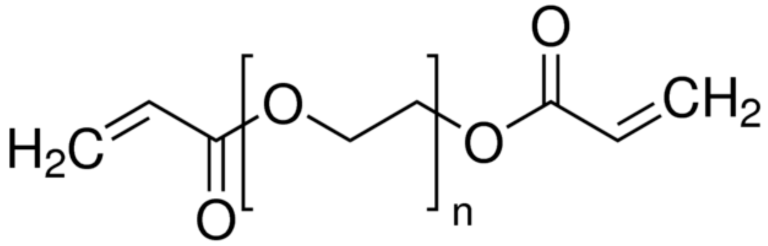

Figure S5. Structure of poly (ethylene glycol) diacrylate (PEGDA).

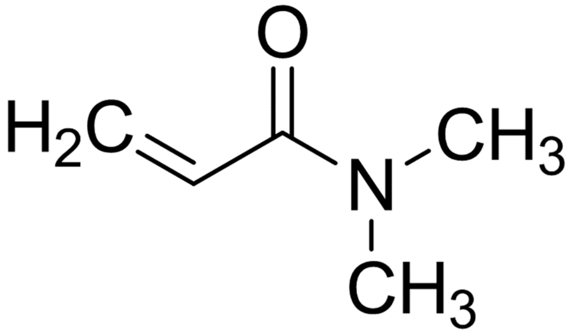

Figure S6. Structure of dimethyl acrylamide (DMAAm).

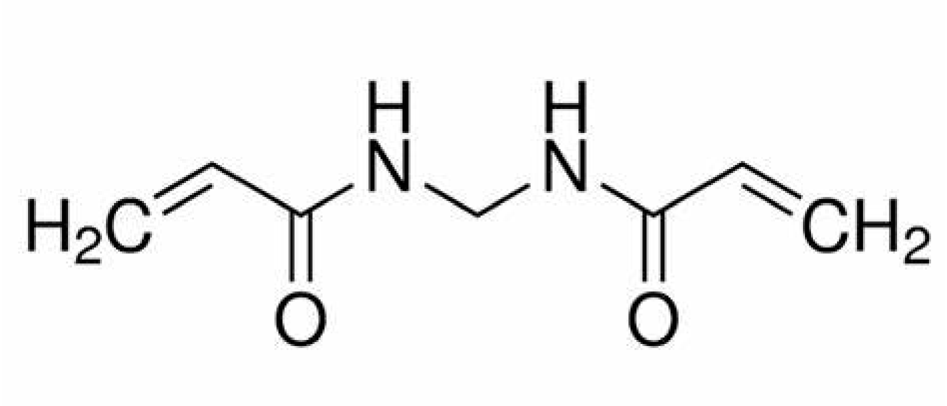

Figure S7. Structure of methylene bis-acrylamide (MBAAm).

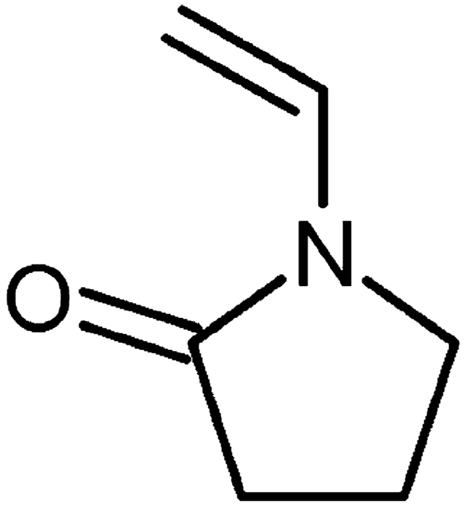

Figure S8. Structure of ninylpyrrolidone (NVP).

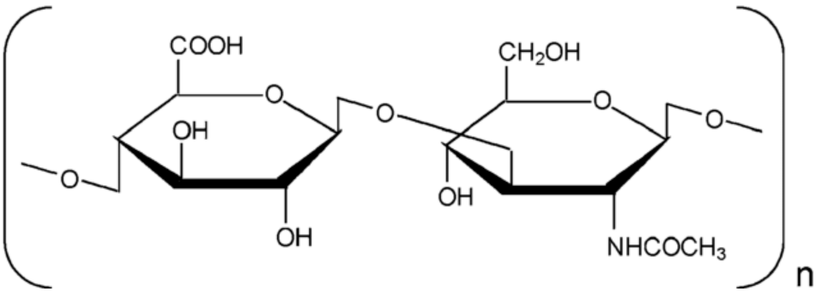

Figure S9. Structure of gelatin[4].

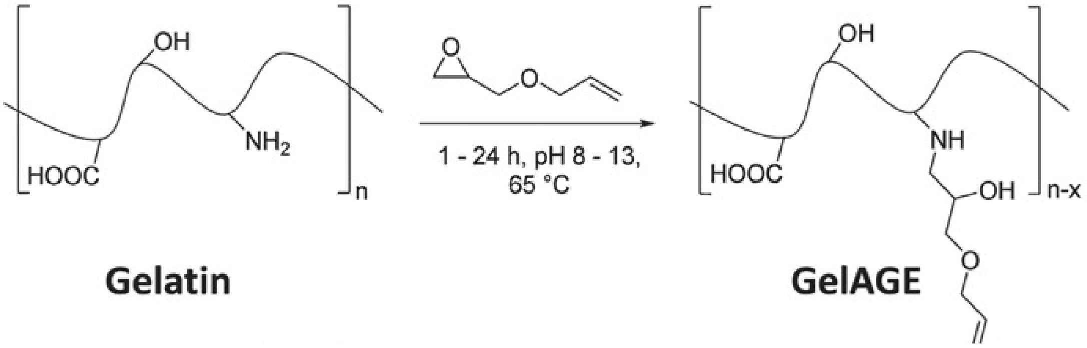

Figure S10. Synthetic routine of allyl glycidyl ether (AGE) modified gelatin [5].

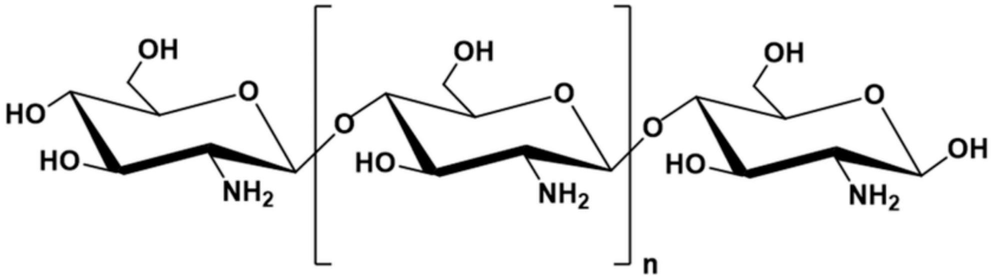

Figure S11. Structure of chitosan.

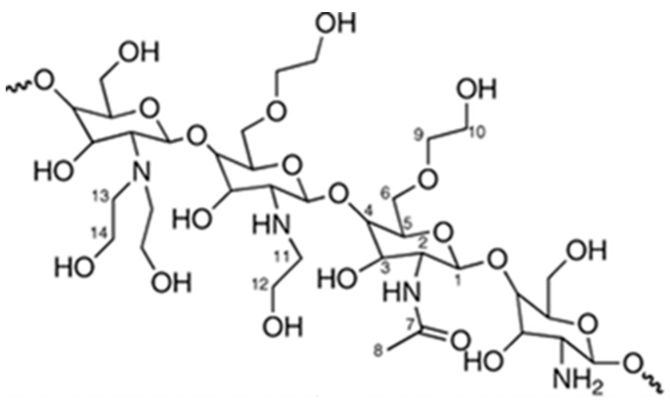

Figure S12. Structure of glycol chitosan (GC) [6].

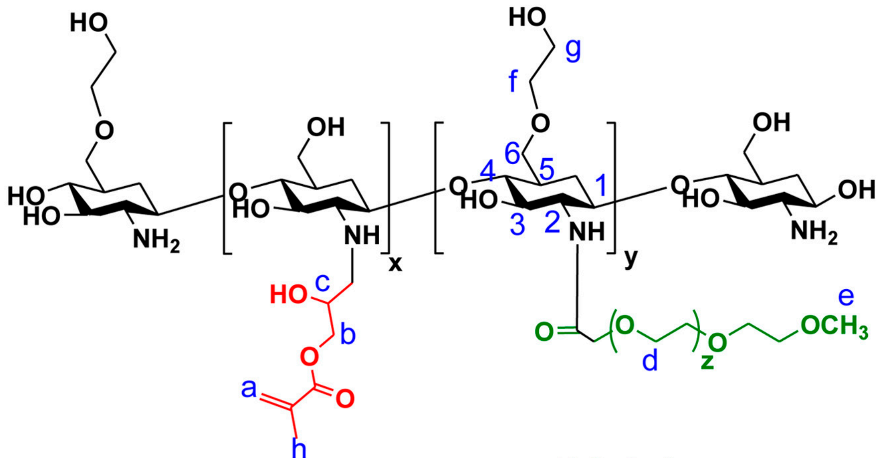

Figure S13. Structure of methacrylated GC (MeGC) [7].

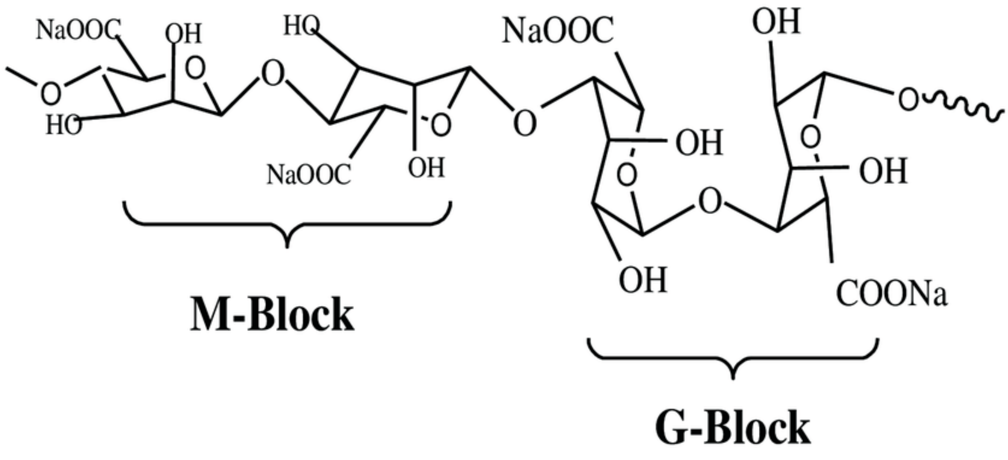

Figure S14. Structure of alginate [8].

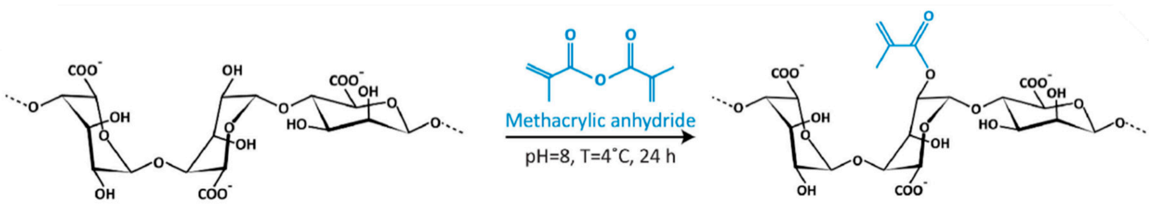

Figure S15. Synthetic routine of methacrylated alginate (Alg-MA) treating the secondary hydroxyl groups with MAA [9].

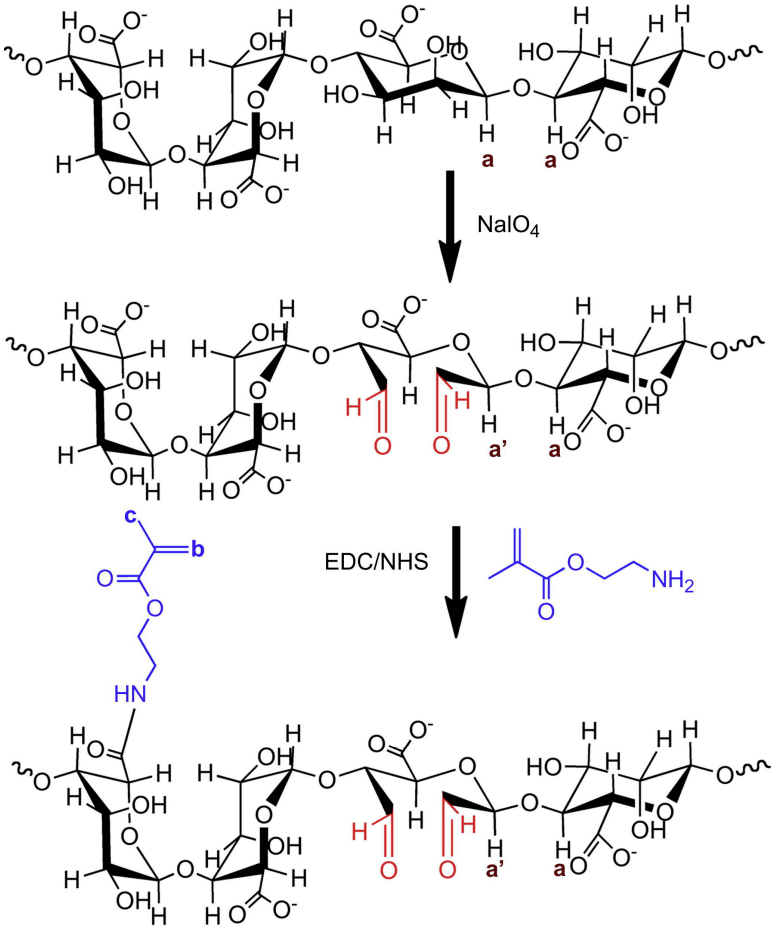

Figure S16. The synthetic routine of oxidized and methacrylated alginates (OMA) [10].

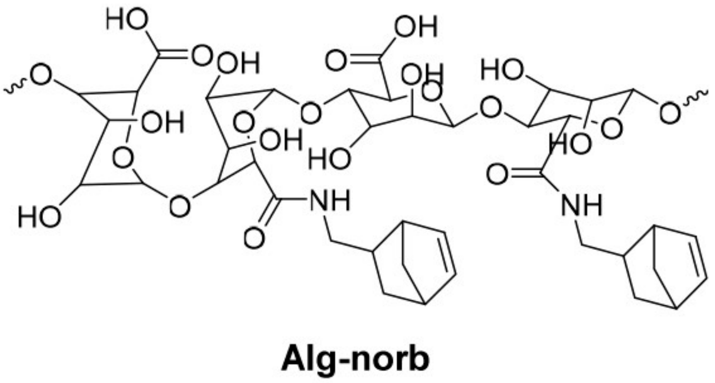

Figure S17. Structure of norbornene functionalized alginate[11].

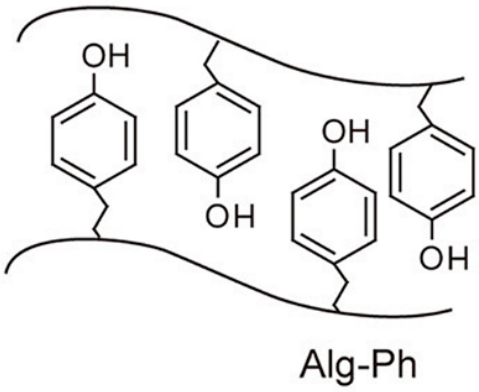

Figure S18. Structure of phenyl group functionalized alginate (Alg-Ph)[12].

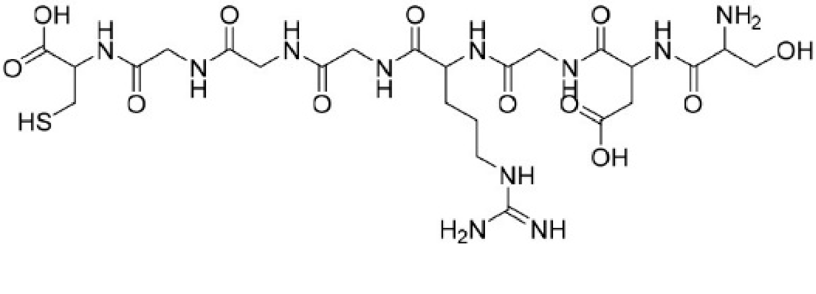

Figure S19. Structure of RGD Peptide Sequence (CGGGRGDS) [11].

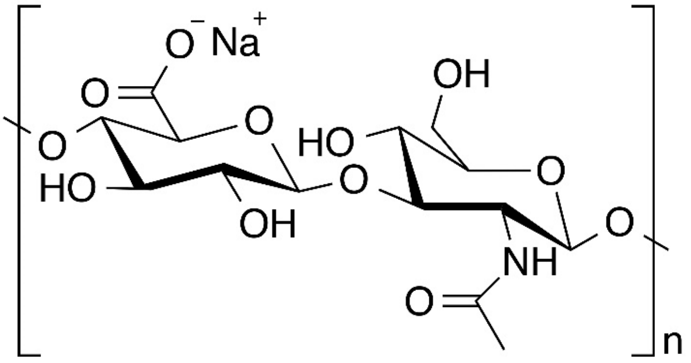

Figure S20. l Structure of hyaluronan N-acetyl-D-glucosamine and β-D-glucuronic acid linked by β-1,3 and β-1,4 glycosidic bonds.

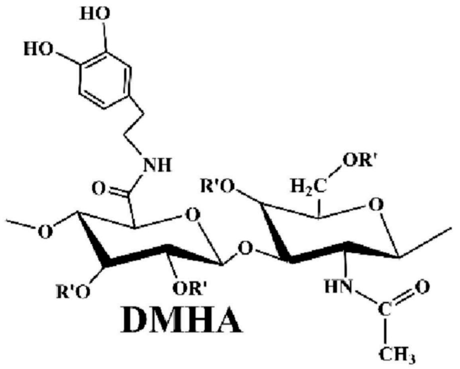

Figure s21. Structure of dopamine-conjugated maleic hyaluronic acid (DMHA) [13].

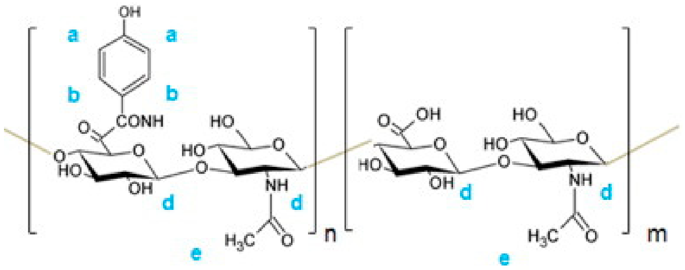

Figure S22. Structure of tyramine-functionalized hyaluronic acid (HA-Tyr) [14].

References

1. Wan, T.; Fan, P.; Zhang, M.; Shi, K.; Chen, X.; Yang, H.; Liu, X.; Xu, W.; Zhou, Y., Multiple

Crosslinking Hyaluronic Acid Hydrogels with Improved Strength and 3D Printability. *ACS Appl Bio Mater* **2022,** 5, (1), 334-343.

2. Vigata, M.; Meinert, C.; Pahoff, S.; Bock, N.; Hutmacher, D. W., Gelatin Methacryloyl Hydrogels Control the Localized Delivery of Albumin-Bound Paclitaxel. *Polymers (Basel)* **2020,** 12, (2).
3. Zanon, M.; Chiappone, A.; Garino, N.; Canta, M.; Frascella, F.; Hakkarainen, M.; Pirri, C. F.; Sangermano, M., Microwave-assisted methacrylation of chitosan for 3D printable hydrogels in tissue engineering. *Materials Advances* **2022,** 3, (1), 514-525.
4. Picard, J.; Giraudier, S.; Larreta-Garde, V., Controlled remodeling of a protein-polysaccharide mixed gel: examples of gelatin-hyaluronic acid mixtures. *Soft Matter* **2009,** 5, (21).
5. Bertlein, S.; Brown, G.; Lim, K. S.; Jungst, T.; Boeck, T.; Blunk, T.; Tessmar, J.; Hooper, G. J.; Woodfield, T. B. F.; Groll, J., Thiol-Ene Clickable Gelatin: A Platform Bioink for Multiple 3D Biofabrication Technologies. *Adv Mater* **2017,** 29, (44), 1703404.
6. Amsden, B. G.; Sukarto, A.; Knight, D. K.; Shapka, S. N., Methacrylated glycol chitosan as a photopolymerizable biomaterial. *Biomacromolecules* **2007,** 8, (12), 3758-66.
7. Yang, D. H.; Seo, D. I.; Lee, D.-W.; Bhang, S. H.; Park, K.; Jang, G.; Kim, C. H.; Chun, H. J., Preparation and evaluation of visible-light cured glycol chitosan hydrogel dressing containing dual growth factors for accelerated wound healing. *Journal of Industrial and Engineering Chemistry* **2017,** 53, 360-370.
8. Salisu, A.; Sanagi, M. M.; Abu Naim, A.; Wan Ibrahim, W. A.; Abd Karim, K. J., Removal of lead ions from aqueous solutions using sodium alginate-graft-poly(methyl methacrylate) beads. *Desalination and Water Treatment* **2015,** 57, (33), 15353-15361.
9. Hasany, M.; Talebian, S.; Sadat, S.; Ranjbar, N.; Mehrali, M.; Wallace, G. G.; Mehrali, M., Synthesis, properties, and biomedical applications of alginate methacrylate (ALMA)-based hydrogels: Current advances and challenges. *Applied Materials Today* **2021,** 24.
10. Jeon, O.; Alt, D. S.; Ahmed, S. M.; Alsberg, E., The effect of oxidation on the degradation of photocrosslinkable alginate hydrogels. *Biomaterials* **2012,** 33, (13), 3503-14.
11. Ooi, H. W.; Mota, C.; Ten Cate, A. T.; Calore, A.; Moroni, L.; Baker, M. B., Thiol-Ene Alginate Hydrogels as Versatile Bioinks for Bioprinting. *Biomacromolecules* **2018,** 19, (8), 3390-3400.
12. Sakai, S.; Komatani, K.; Taya, M., Glucose-triggered co-enzymatic hydrogelation of aqueous polymer solutions. *RSC Adv.* **2012,** 2, (4), 1502-1507.
13. Fan, P.; Dong, Q.; Yang, J.; Chen, Y.; Yang, H.; Gu, S.; Xu, W.; Zhou, Y., Flexible dual-functionalized hyaluronic acid hydrogel adhesives formed in situ for rapid hemostasis. *Carbohydr Polym* **2023,** 313, 120854.
14. Loebel, C.; D'Este, M.; Alini, M.; Zenobi-Wong, M.; Eglin, D., Precise tailoring of tyramine-based hyaluronan hydrogel properties using DMTMM conjugation. *Carbohydrate Polymers* **2015,** 115, 325-333.
